# Exploratory dynamics of vocal foraging during infant-caregiver communication

**DOI:** 10.1038/s41598-020-66778-0

**Published:** 2020-06-26

**Authors:** V. P. S. Ritwika, Gina M. Pretzer, Sara Mendoza, Christopher Shedd, Christopher T. Kello, Ajay Gopinathan, Anne S. Warlaumont

**Affiliations:** 1 University of California, Merced, Department of Physics, Merced, CA 94343 USA; 2 University of California, Merced, Cognitive and Information Sciences, Merced, CA 95343 USA; 3 University of California, Los Angeles, Department of Communication, Los Angeles, CA 90095 USA

**Keywords:** Social behaviour, Human behaviour

## Abstract

We investigated the hypothesis that infants search in an acoustic space for vocalisations that elicit adult utterances and vice versa, inspired by research on animal and human foraging. Infant-worn recorders were used to collect day-long audio recordings, and infant speech-related and adult vocalisation onsets and offsets were automatically identified. We examined vocalisation-to-vocalisation steps, focusing on inter-vocalisation time intervals and distances in an acoustic space defined by mean pitch and mean amplitude, measured from the child’s perspective. Infant inter-vocalisation intervals were shorter immediately following a vocal response from an adult. Adult intervals were shorter following an infant response and adult inter-vocalisation pitch differences were smaller following the receipt of a vocal response from the infant. These findings are consistent with the hypothesis that infants and caregivers are foraging vocally for social input. Increasing infant age was associated with changes in inter-vocalisation step sizes for both infants and adults, and we found associations between response likelihood and acoustic characteristics. Future work is needed to determine the impact of different labelling methods and of automatic labelling errors on the results. The study represents a novel application of foraging theory, demonstrating how infant behaviour and infant-caregiver interaction can be characterised as foraging processes.

## Introduction

## Infant vocal development

Human infants show massive growth in vocalising abilities during their first year^1–5^. Cries and short, quiet sounds dominate early vocalisations. By about three months, infants demonstrate a much wider range of vocalisation types, varying pitch, amplitude, and other phonatory characteristics. During this time, they also start producing primitive consonant-vowel articulations. By 7 months, infants begin producing well-timed adult-like consonant-vowel alternations. This expansion in repertoire lays a foundation for later speech and other vocal communication production^2,6^. For instance, there is continuity between the sounds produced during prelinguistic stages of vocal development and those sounds that make up infants’ first words^7^.

Although anatomical changes and neuromaturation may account for some of the changes during the first year, even newborn infant vocal tracts are capable of producing a very wide range of sounds, and the dramatic changes in infants’ vocalisations over the first year are believed to be primarily due to learning^8–10^. Computational models of infant vocal learning have demonstrated that some combination of exploratory processes, social or intrinsic rewards, and imitation of adult speech input can result in the vocal learning we see in human infancy^11–17^. Infant vocal learning may be viewed as a process that combines variation and selection, resulting in the evolution of a more adult-like repertoire of sound types. This perspective raises the question of how variability serves to explore the space of vocalisations in a way that selects those that are successful in terms of social interaction and communication.

A large body of research with human infants provides strong support for the role of social responses in shaping infant vocal learning at multiple timescales. Adult responses are said to be contingent on infant vocalisation type if a response is more likely for some types of vocalisations than for other types. Adult responses contingent on speech-related infant vocalisations result in more frequent speech-related and adult-like vocalisations by the infant during the seconds and minutes following the adult response^10,18–22^. At the longer timescales of months and years, differences in adult responses predict later communication abilities^23–25^. Differences in feedback loops wherein infant vocalisations generate adult responses that in turn impact future infant vocalisations appear to underlie some of the differences in speech development that are observed on average across socioeconomic and clinical groups^21,26,27^.

Less is known about exploratory dynamics in the space of sounds produced by vocalisations. Research has documented temporal clustering in the occurrence of different prelinguistic vocalisation types, termed “session effects” because the effect is that one recording session can provide a very different view of an infant’s vocal repertoire than one made a few minutes later^28^. Moreover, infant vocalisations have been found to be clustered across a wide range of timescales, with bouts of infant vocalisations clustering within larger episodes of high-volubility with those in turn clustered over even longer time spans^29^. The same study also found that the adult vocalisations to which infants were exposed also exhibited nested temporal clustering, with the degree of nesting being coordinated between infants and adults.

The finding of coordinated clustering in the timing of adult vocalisations, along with the observed dependencies between infant and adult vocalisations, show adults to be active agents in infant speech development. Indeed, a large volume of prior research has documented how adults alter the timing, acoustic properties, and content of their vocalisations when they are directed to an infant^30–35^, including the nested temporal clustering of their speech sounds^36^. While less research has focused on how future adult behaviour is influenced by infant responses to adult vocalisations, we do know that infant vocalisations tend to follow adult vocalisations during naturalistic interactions^37–39^ and that infant responses are sensitive to differences in adult behaviours^40^. Taken together, these findings suggest that both infants and adults may be exploring their vocalisation spaces for sounds that elicit social engagement.

## Infant-caregiver communication as foraging

The idea of searching a space of vocalisations for social rewards has parallels with foraging in natural environments and other domains as studied in the behavioural and cognitive sciences. Humans and other animals forage physical spaces to collect food, identify mates or find safe locations to escape predators. Similarly, experiments on memory and perceptual search have shown that foraging dynamics in physical spaces also play out in semantic and visual spaces^41–45^.

In all the domains above, search dynamics have been found to exhibit a common pattern hypothesized to reflect an optimally efficient balance of intensive versus extensive excursions^46^. In many cases, the resulting strategy can be characterized by a Lévy walk, which is a random search strategy consisting of mostly short steps interspersed with a “heavy tail” of long steps, characterised by step lengths *l* occurring with a power law probability distribution $P(l)\sim {l}^{-\alpha }$ with $1 < \alpha \le 3$. Lévy walks are effective when resources are scarce or patchy^46^, and they are parameterised by a scaling exponent, *α*, that has been shown to adapt to the distribution of resources^47^. Depending on the features of the resource distribution, step lengths have also been modelled by lognormal distributions, defined as $P(l)={(l\sigma \sqrt{2\pi })}^{-1}{e}^{-{(\text{ln}l-\mu )}^{2}/2{\sigma }^{2}}$ which have “lighter” tails than the power law^48^. Finally, when resources are more plentiful, the distribution of step lengths has been modelled in terms of exponential decay, $P(l)\sim {e}^{-\lambda l}$, where the likelihood of a given step size drops off much more rapidly than a power law as step size increases^49^. Thus, foraging behaviours are generally adaptive to their environments and resource distributions and therefore, studying them can yield insights into the distribution and dynamics of resources.

The literature on foraging suggests that it may be fruitful to characterise infant-caregiver vocalisations in terms of steps that are distributed over a space of possible sounds. A similar approach was taken in a previous study of infant eye movements as a window into audio-visual speech perception that reveals differences in processing for infants with autism spectrum disorder^50^. Given the adaptive nature of search patterns found in previous studies, we can hypothesise that infant and/or adult vocalisation strategies, in terms of the dynamics of how they are clustered in time and how they explore the landscape of possible acoustic features, may change over the course of language and social development, as infants gain increasing mastery over their vocal motor systems and learn about the consequences of their vocal behaviours, and as adults adapt their behaviours to accommodate infant communication^29^. Vocal foraging may be expressed in different representations of the sound space, and it may also be expressed in the temporal dynamics of sound production, whereby time serves as a proxy for search in a mental space^42,43^). Indeed, in one of the first studies to report Lévy walks in animal foraging, time steps between foraging events were used as a proxy for measuring distances traversed in physical space^51^.

## The present study

Here we study the dynamics of socially-guided vocal exploration over the course of a day by infants and their caregivers from a foraging perspective. We assess exploration in terms of patterns of movement in an acoustic space, an approach which has not previously been employed in the study of infant vocalisations and which will allow comparisons with other foraging behaviours.

We utilise automatically labelled day-long audio recordings, which have two main advantages. First, they provide ecologically valid samples of infant and adult behaviour in the range of contexts typically experienced by the infant. Second, they provide long time series with large numbers of events, which is critical in order to characterise the distributions of step lengths (i.e. acoustic distances or time intervals between successive vocalisations), particularly the tails of those distributions.

We define acoustic space in terms of two dimensions, the mean pitch and amplitude of each vocalisation. We chose these dimensions for this initial study because they are relatively easy to measure, reflect aspects of intonation, emphasis, intention, and emotional valence that are integral to verbal communication, and are features that appear to be actively explored by infants starting from the earliest months of life^28,30,31,39^. It should be noted that these acoustic dimensions are measured from the infant’s perspective, since the audio recordings come from infant-worn recorders. In the case of adult amplitude measurements, this creates a situation in which the data represent amplitude as heard by the child, which will vary as a function of both amplitude as produced by the adult’s vocal tract and proximity of the adult to the child. Recent research has shown this amplitude as heard by the infant to vary as a function of whether adult speech is directed to the target child or not^39^, and it is a real and likely salient acoustic feature from the child’s perspective. Note that the pitch dimension is not expected to be influenced by proximity of the adult to the child.

The focus is on step sizes, measured through inter-vocalisation intervals and vocalisation-to-vocalisation acoustic differences. Inter-vocalisation intervals could potentially serve as proxies for acoustic or mental distance, as in previous foraging studies. Thus, our first analyses focus on whether there is a correlation between inter-vocalisation time interval and inter-vocalisation acoustic difference.

Next we ask whether infant and caregiver vocalisation-to-vocalisation step sizes change with infant age and depending on whether the speaker’s prior vocalisation received a response. Our interest in changes in exploration with age stems from the fact that over the first 18 months of life (the range of ages included in our datasets) infants exhibit large developmental changes in the types of vocalisations they produce and in their ability to use vocalisation for a range of communicative purposes.

If vocalisations are like search movements that may ‘find’ rewards as expressed by vocal responses, then intervals should be shorter after rewards i.e. vocal responses. This prediction is based on prior foraging studies showing that humans and other animals tend to restrict search movements after finding resources, presumably in the hopes of finding more nearby, a phenomenon referred to as ‘area-restricted search’^47^. As was reviewed above, prior work has found evidence of bi-directional contingencies in the inter-response timing of infant and adult vocalisations, that adults are especially likely to respond vocally to infant vocalisations that are more advanced, that infants’ subsequent vocalisations are shaped by recent adult responses, and that adults actively modify their vocalisation acoustics when they address an infant versus an adult. Given these prior findings, we would expect that, seen from a foraging perspective, infants’ and adults’ inter-vocalisation step sizes would be smaller (both in time and acoustically) when the vocaliser has just received a social response. This would be evidence that the vocalisers are engaged in a foraging exploration-exploitation process wherein vocal responses are serving as a type of reward.

Finally, we test whether there are particular acoustic properties or patterns of movement in acoustic space that predict that an infant vocalisation will be followed by an adult vocalisation, and vice versa.

## Results

### Steps in time and acoustic space

**Figure 1:**
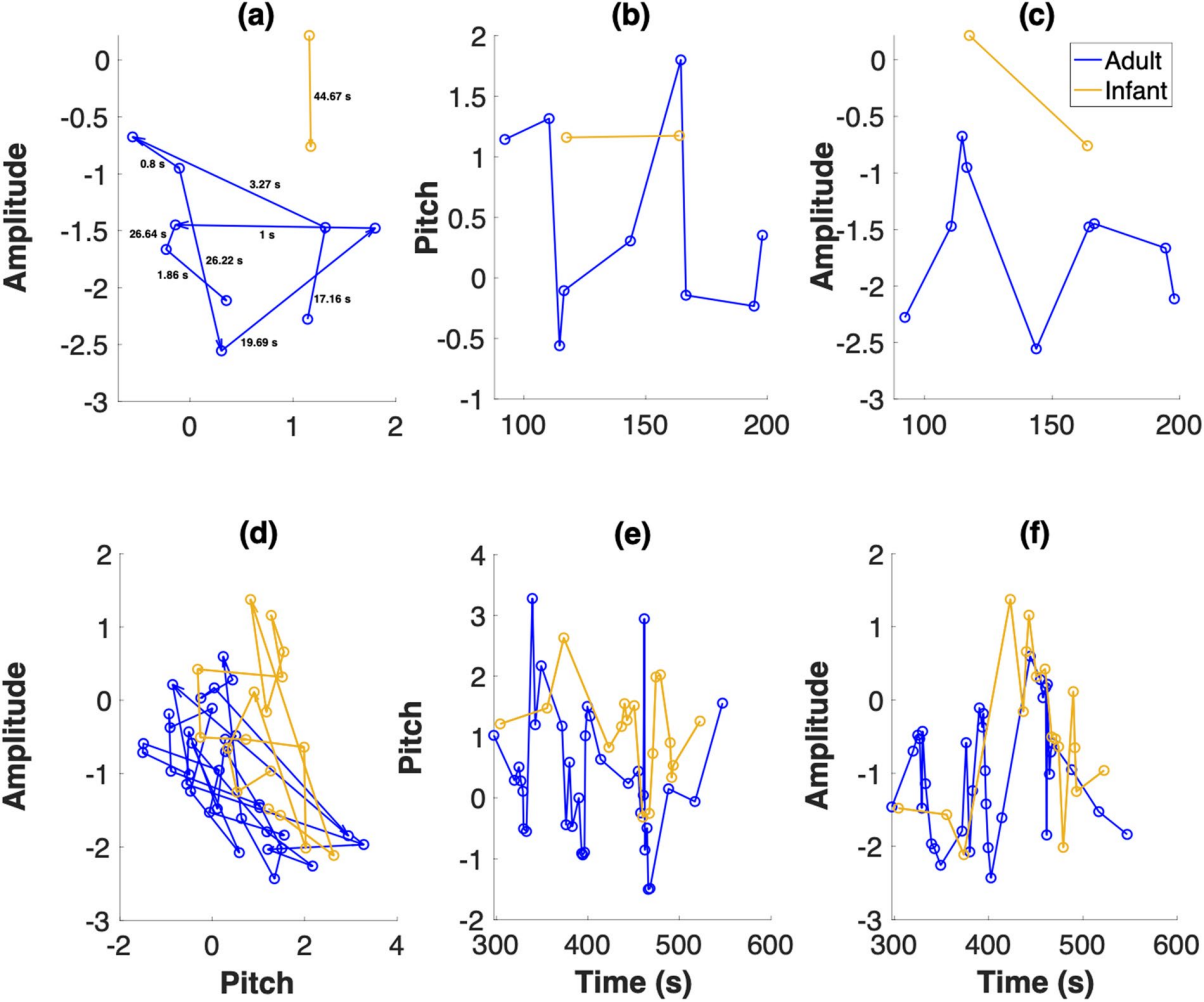
Sample vocalisation sequences by a 3-month-old infant and the infant’s caregiver(s). (**a**) shows the movement of the infant (yellow) and adult (blue) in a 2D acoustic space defined by mean pitch and amplitude. Directed arrows indicate the direction of the vocaliser’s movement in the acoustic space. This can be thought of as a foraging process-like depiction. The inter-vocalisation time intervals are indicated. (**b**,**c**) Show the same data with time plotted on the X axis, and pitch and amplitude respectively on the Y axis. In (**a**–**c**), a vocalisation by the adult is indicated by a blue open circle, and a vocalisation by the infant is indicated by a yellow open circle. Note that these are the same data that is depicted in Fig. 2. (**d**–**f**) Show plots for a longer period of time taken from the same recording as in (**a**–**c**).

**Figure 2:**
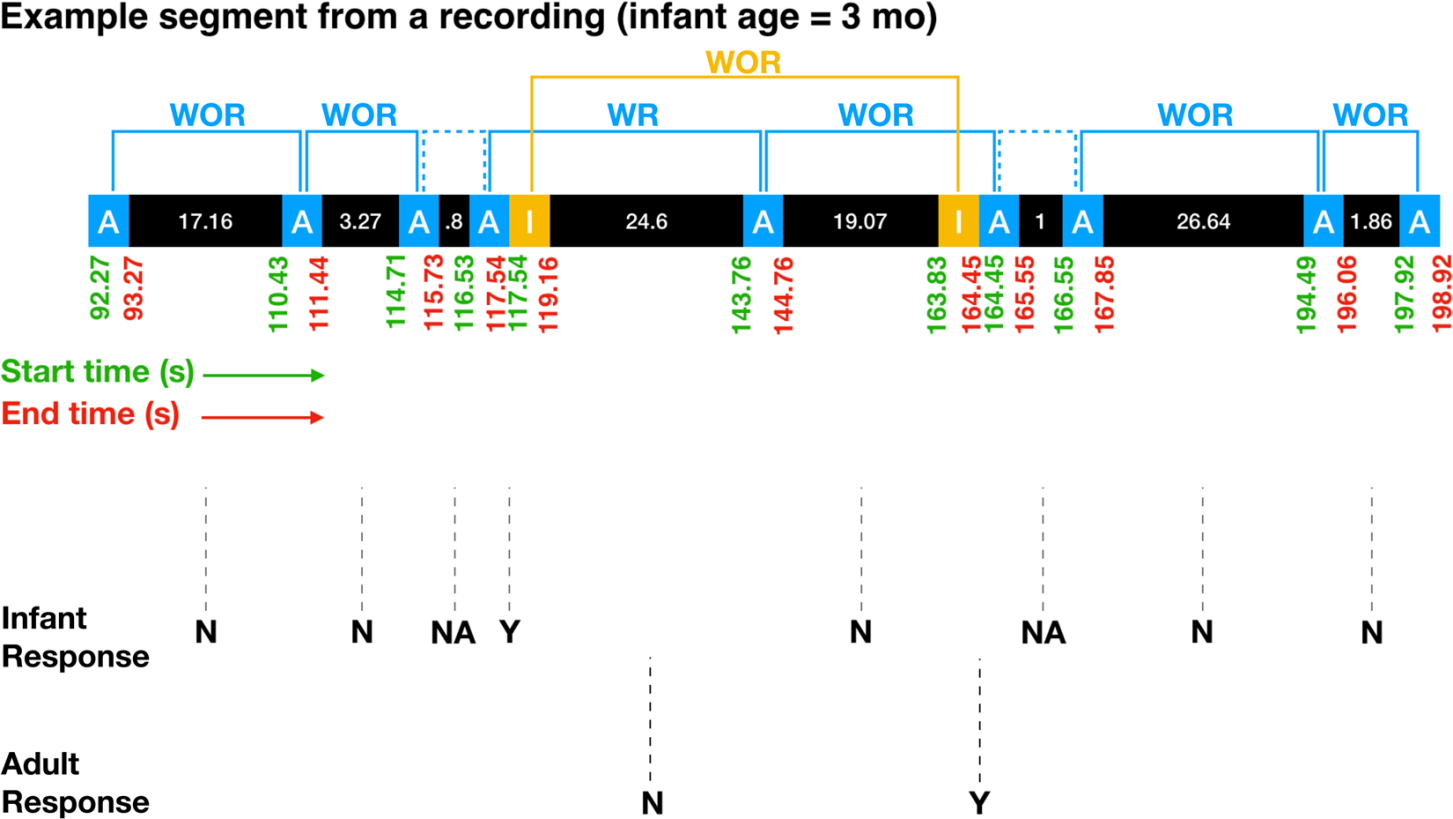
Illustration of the automatic labelling scheme. Here, we use a small portion from a recording of an infant at age 3 months to illustrate the labelling scheme. The information in the top portion of the figure is provided by the LENA system; inter-vocalisation intervals, acoustic features, and response codes are computed automatically based on LENA-generated speaker labels and the original audio file. Adult vocalisations are in blue boxes while infant vocalisations are in yellow boxes. The start time of each vocalisation is indicated in green below the corresponding box, on the left, while the end time is in red and towards the right of the box. Inter-vocalisation intervals (in s) are given in black boxes between subsequent vocalisations, and the length of the box is indicative of the duration of the inter-vocalisation interval. For each infant/adult vocalisation, the receipt of a response (Y/N/NA) is indicated (dotted vertical line). An adult vocalisation is labelled as having been followed by an infant “response” (WR for “with response”; Y in the Infant Response row) if the onset of an infant vocalisation occurs within 1 s following the offset of the adult vocalisation, with no intervening adult vocalisation. The same logic applied for labelling whether adult “responses” followed infant vocalisations. If two adult vocalisations are separated by 1 s or less without an intervening infant vocalisation, then response to the first is NA. Adult vocalisation steps analysed in this study are indicated by the blue lines, with steps following a response labelled WR in blue, and steps following no response labelled WOR (for “without response”) in blue. Infant vocalisation steps are indicated by the yellow lines, with steps following a response labelled WR in yellow, and steps following no response labelled WOR in yellow. Dotted lines connecting vocalisations indicate that the corresponding inter-vocalisation interval was not analysed because it was less than or equal to 1 s with no intervening response. Analysed inter-vocalisation intervals were divided into WR and WOR depending on whether the first vocalisation in the sequence was followed within 1 s by a “response”.

Figure 1 presents an example of infant vocalisation steps in 2D acoustic space as a function of recording time. Euclidean distance in the acoustic space is defined as $s=\sqrt{\Delta {f}^{2}+\Delta {d}^{2}}$, where Δ*f* is the change in mean fundamental frequency (log f_0_) from the ${i}^{th}$ to $i+{1}^{th}$ vocalisation, and Δ*d* is the corresponding change in mean amplitude (note that acoustic dimensions have been standardised). Steps for each speaker type were divided into two groups (Fig. 2): (1) steps following receipt of a response by the other speaker type (referred to as WR, for ‘with response’) and (2) steps following a vocalisation that did not receive a response (referred to as WOR, for ‘without response’).

We found weak positive correlations between distance in 2-D acoustic space (i.e., the step size from the ${i}^{th}$ to $i+{1}^{th}$ vocalisation in acoustic space) and the corresponding inter-vocalisation time intervals for both infants (over the 143 recording days, mean $r=0.10$, mean $p=0.11$, median $r=0.09$, median $p=0.01$, mean $n=1043.86$, where $r$ is the Pearson correlation coefficient and $p$ is the probability of the null hypothesis) and adults (mean $r=0.08$, mean $p=0.06$, median $r=0.08$, median $p < 0.001$, mean $n=3223.40$). Note that the number of adult vocalisation events is much larger than that of infant vocalisation events. Results from a linear mixed effects model with acoustic step size being predicted by inter-vocalisation interval, controlling for age and infant ID, indicate that step sizes in acoustic space and time are positively correlated for both infants (*β* = 0.07, p = <0.001, n = 149,272, where *β* is the standardized regression coefficient) and adults (*β* = 0.05, p = <0.001, n = 460,946). These results also hold for acoustic step sizes and inter-vocalisation intervals following the reception of a response (WR) and following vocalisations that did not receive responses (WOR). For more details, see Supplementary Table S4.

At the recording day level, step sizes in 2-D acoustic space were found to be predominantly lognormal for both infant and adult vocal exploration for both step types, based on Akaike information criterion (AIC)^52^. Step sizes in individual acoustic dimensions were exponentially distributed for all step types for both speaker types, per AIC (Fig. S4, Supplementary Information). Inter-vocalisation intervals between adult vocalisations were predominantly Pareto distributed for both step types. A Pareto distribution is a type of power law distribution given by $P(x)=\frac{\alpha {x}_{min}^{\alpha }}{{x}^{\alpha +1}}$, where $x\in [{x}_{min},\infty ]$. If the exponent $\alpha$ is such that $1 < \alpha \le 3$, then a Pareto distribution can be used to describe a Lévy walk. Inter-vocalisation times between infant vocalisations were lognormally distributed for both step types. The unsplit step size distributions (i.e., step size distributions that were not split into WR and WOR categories) were largely of the same type as the split step size distributions, except for unsplit time step sizes between adult vocalisations, which were found to be predominantly lognormal (Fig. S4). For all step size distribution analyses comparing WR to WOR, we only considered steps for which the corresponding inter-vocalisation interval was at least 1 s. This was to control for the fact that determining a response was not received required a wait of 1 s (see Methods).

A randomly selected example of the probability distributions obtained from the day-level recording data compared to the corresponding AIC fit is shown in Fig. S3. We see reasonable agreement between the two based on visual observation. In addition, we calculated the coefficient of determination (R^2^) for all fits computed using AIC and generally found good agreement between raw data and fits. R^2^ values indicated especially good fits for WOR and unsplit step size distributions (which had larger sample sizes compared to WR step size distributions), and the mean R^2^ value for all fits was found to be 0.73 (Table S5).

There was no statistically significant interaction between response and age on any of the step size measures. Thus, we do not include an interaction term in any of the statistical tests presented below. For results from statistical tests from models with interaction between response and age effects as a predictor see https://osf.io/8ern6/.

### Changes with age

**Figure 3:**
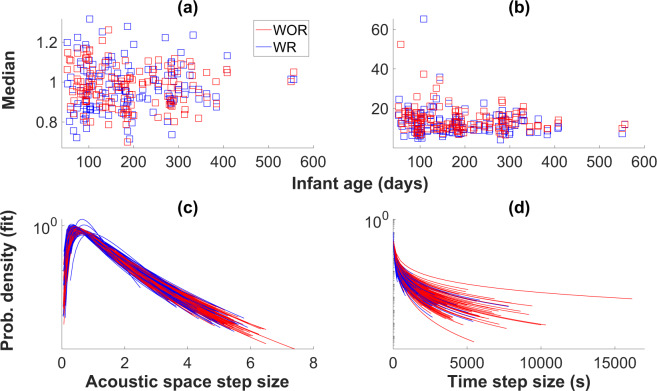
Median values and fitted distributions for acoustic space step size and inter-vocalisation interval distributions for infants. (**a**) Median values of infant acoustic step size distributions split into steps following an adult response (WR, blue), and steps following vocalisations that did not receive adult responses (WOR, red). (**b**) Median values of infant inter-vocalisation interval distributions for WR and WOR steps. Median values were computed based on data, not AIC best fits. (**c**,**d**) Infant acoustic step size and inter-vocalisation interval distributions, respectively, fit to lognormal distributions, based on AIC, are shown in log-linear plots. Only distributions that are best fit to lognormal are shown.

Median infant step size in the 2D acoustic space increased significantly with infant age (Table 1 and Fig. 3; see also Table S8 and Fig. S10). Infant step size in the pitch dimension increased as infants got older (Table S7 and Fig. S7). However, infant step size in the amplitude dimension if anything got smaller as infants got older (Table S6 and Fig. S5). There were no statistically significant effects of age on infant inter-vocalisation time intervals (Table 1 and Fig. 3; see also Supplementary Table S9 and Fig. S12).

**Table 1:** Results of mixed effects regressions predicting median and 90^th^ percentile values of vocal foraging measures, as well as parameters of probability distributions of vocal foraging measures, as a function of whether a response was recently received, infant age, and sample size, with infant ID as a random effect.

Dependent variable	Response effect	Age effect	Sample size effect
Inf. acoustic step size (median)	−0.02 (p = 0.85)	**0.15 (p** = **0.02)**	−**0.31 (p** < **0.001)**
Inf. acoustic step size (90^th^ perc.)	−0.18 (p = 0.13)	0.09 (p = 0.14)	−**0.24 (p** = **0.002)**
Inf. acoustic step size (lognormal *μ*)	0.01 (p = 0.93)	0.12 (p = 0.06)	−**0.30 (p** < **0.001)**
Inf. acoustic step size (lognormal *σ*)	−**0.41 (p** < **0.001)**	0.02 (p = 0.70)	**0.18 (p** = **0.04)**
Inf. inter-voc. interval (median)	−**0.25 (p** = **0.01)**	0.06 (p = 0.22)	−**0.62 (p** < **0.001)**
Inf. inter-voc. interval (90^th^ perc.)	−0.11 (p = 0.24)	0.004 (p = 0.94)	−**0.57 (p** = **0.01)**
Inf. inter-voc. interval (lognormal *μ*)	−**0.19 (p** = **0.01)**	0.03 (p = 0.52)	−**0.78 (p** < **0.001)**
Inf. inter-voc. interval (lognormal *σ*)	−**0.43 (p** < **0.001)**	0.01 (p = 0.76)	−**0.76 (p** < **0.001)**
Ad. acoustic step size (median)	**0.24 (p** = **0.01)**	**0.39 (p** < **0.001)**	0.07 (p = 0.23)
Ad. acoustic step size (90^th^ perc.)	0.02 (p = 0.83)	**0.29 (p** < **0.001)**	**0.19 (p** = **0.01)**
Ad. acoustic step size (lognormal *μ*)	**0.26 (p** = **0.003)**	**0.46 (p** < **0.001)**	0.05 (p = 0.47)
Ad. acoustic step size (lognormal *σ*)	−**0.61 (p** < **0.001)**	−**0.16 (p** = **0.01)**	0.12 (p = 0.11)
Ad. inter-voc. interval (median)	**0.16 (p** = 0.05)*	**0.22 (p** < **0.001)**	−**0.45 (p** < **0.001)**
Ad. inter-voc. interval (90^th^ perc.)	−0.07 (p = 0.46)	0.04 (p = 0.43)	−**0.50 (p** < **0.001)**
Ad. inter-voc. interval (pareto *x*_*min*_)	**0.65 (p** < **0.001)**	−0.003 (p = 0.96)	−0.04 (p = 0.50)
Ad. inter-voc. interval (pareto *α*)	**0.30 (p** < **0.001)**	−**0.24 (p** < **0.001)**	**0.37 (p** < **0.001)**

The *β* values are given with the *p* values in brackets. Significant effects (at a significance level of 0.05) are indicated in bold. All values reported have been rounded to two decimal points wherever possible.

*The p-value here is actually 0.0479 and has been rounded to 0.05, which is why it is in bold, indicating significance.

The median as well as the 90^th^ percentile value of the adult vocal step distributions increased with infant age (see Table 1) which is in agreement with the increase in the *μ* of the lognormal fits to adult acoustic step size distributions as infant age increased and with the decrease in *σ* (see Fig. S9a). An increase in the *μ* parameter is associated with a wider, right-shifted peak and a wider tail, making both intermediate steps and larger steps more likely. On the other hand, as *σ* increases, the peak and the tail widens, with the peak also shifting to the left. This makes shorter and larger steps more likely. The changes were evident in both the amplitude and the pitch dimensions (Tables S6 and S7). Median inter-vocalisation intervals increased significantly, which is consistent with the decrease in inter-vocalisation interval Pareto *α* values. As *α* increases, the distribution decays rapidly and the likelihood of larger step sizes decreases. (For a demonstration of how lognormal and pareto distributions change as a function of their parameters, see Supplementary Section 3.3 as well as https://osf.io/2fuje/). Note that for the analyses of parameters based on step-size distribution fits, we only used those distributions that were best fit to the predominant fit for that distribution type. For example, for *μ* or *σ* parameters of adult vocal step distributions, only those distributions that were best fit to lognormal were analysed.

### Infant step sizes after receiving an adult response

As shown in Table 1 and Fig. 3, infants’ median and 90^th^ percentile acoustic space step sizes did not show statistically significant differences as a function of whether the first vocalisation received a response (see also Table S8 and Fig. S10). Upon analysing the parameters of the lognormal fits, we found a smaller *σ* when a response was received after the first vocalisation in a step. A small *σ* is associated with a right-shifted peak as well as a smaller tail to the distribution (Fig. S9a). When looking individually at steps in amplitude (Table S6, Fig. S5) and steps in pitch (Table S7, Fig. S7), steps in amplitude showed no statistically significant differences as a function of response, and steps in pitch showed only a significantly increased median step size for post-response steps.

Infant inter-vocalisation time intervals were shorter following an adult response, as indicated by statistically smaller median, *μ*, and *σ* values (Table 1, Fig. 3, Table S9, and Fig. S12). Infants vocalised again more quickly following the receipt of an adult response.

### Adult step sizes after receiving an infant response

**Figure 4:**
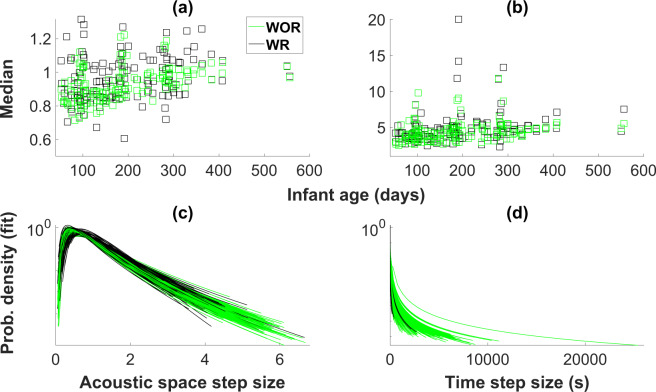
Median values and fitted distributions for acoustic space step size and inter-vocalisation interval distributions for adults. (**a**) Median values of adult acoustic step size distributions split into WR (black) and WOR (green). (**b**) Median values of adult inter-vocalisation interval distributions for WR and WOR. Median values were computed based on data, not AIC best fits. (**c**,**d**) Adult acoustic step size and inter-vocalisation interval distributions, respectively. Acoustic step size distributions are fit to lognormal and inter-vocalisation interval distributions are fit to pareto, based on AIC, are shown in log-linear plots. Only distributions that are best fit to lognormal and pareto, respectively are shown.

For adult vocalisations, the median acoustic step size was significantly larger after an infant response was received compared to other acoustic step sizes, as were *μ* values (Table 1, Fig. 4, Table S8, and Fig. S10). On the other hand, *σ* values were significantly smaller for post-response steps. Lower sigma values correspond to distributions with right-shifted peaks but smaller tails. We did not find a significant effect of response for adult 90^th^ percentile 2D acoustic space step size values. Analyses treating amplitude and pitch dimensions individually indicated that steps in the amplitude dimensions increased (larger median and 90^th^ percentile values, and smaller *λ* values; Table S6, Fig. S5) following an infant response whereas steps in the pitch dimension decreased (smaller 90^th^ percentile values and larger *λ* values; Table S7, Fig. S7).

Adult inter-vocalisation intervals tended to decrease following an infant response, as evidenced by larger *α* values (Table 1, Fig. 4, Table S9, and Fig. S12).

### What kinds of vocalisations and vocalisation patterns receive responses?

As shown in Table 2, logistic regression analyses found that infant utterances at lower frequencies and higher amplitudes were more likely to receive adult responses. Overall, the probability of receiving adult responses decreased with infant age. For adults, we found that utterances at higher pitch and amplitude were more likely to receive infant responses, and the overall likelihood of an infant response decreased with infant age, although this effect was only marginally significant (Table 2). We also did some preliminary analyses regarding what (non-directional) vocalisation step sizes in pitch, amplitude, and time predict a response. We found the probability of both infants and adults receiving a response increased when a vocalisation was preceded by a larger amplitude step and decreased when a vocalisation was preceded by larger steps in pitch and time (Table S16).

**Table 2:** Results of logistic regression predicting from a vocalisation’s pitch and amplitude whether a response from the other speaker type will follow.

Dependent variable	Pitch effect	Amplitude effect	Age effect
Whether infant received adult response	−**0.32 (***p* < 0.001**)**	**0.17 (***p* < 0.001**)**	−**0.16 (***p* < 0.001**)**
Whether adult received infant response	**0.23 (***p* < 0.001**)**	**0.45 (***p* < 0.001**)**	−**0.01 (***p* = 0.05)*

Infant age was also included as a predictor. Infant ID was included as a random effect. The *β* values are given with the *p* values in brackets. Significant effects (at a significance level of 0.05) are indicated in bold. All values reported have been rounded to two decimal points.

*The p-value here is actually 0.0495 and has been rounded to 0.05.

### Validation using data re-labelled by human listeners

We had four cases where an entire recording’s automatically identified child and adult vocalisations were given speaker type labels by listeners who were blind to the speaker type as labelled by the automated system. We calculated inter-rater reliability measures comparing LENA’s CHNSP, MAN, and FAN labels to human listener CHN, MAN, and FAN labels, excluding utterances that received any other LENA label or any other human listener label. We found a mean percent agreement of 80.3 (std. dev = 6.83) and a mean Kappa value of 0.68 (std. dev = 0.12) (see Table 3). For the data labelled by two human listeners (infant 340, 183 days; listeners L1 and L3), we found a simple percent agreement between those listeners of 0.99 and a Cohen’s Kappa value of 0.99, indicating high human inter-rater reliability. For more details on inter-rater reliability measures, see Supplementary Section S4.1. We also plotted temporal and acoustic step size distributions for human labelled data compared to automatically labelled data (Figs. S14–S17). Visual inspection of these distributions overlaid on each other looked extremely similar, except for CHN WR steps, for which we had very few human-labelled datapoints (see Table S13). In addition, two sample Kolmogorov-Smirnov tests performed to test the validity of the null hypothesis that step distributions computed from human-labelled data and corresponding LENA-labelled data belong to the same distribution failed to reject the null hypothesis in 78 percent of tests (see Table S14). We also compared correlation coefficients between step sizes in acoustic space and inter-vocalisation intervals, for data re-labelled by human listeners and the corresponding data as labelled by LENA, and found good agreement between the two (Fig. S2), with better agreement for the adult data which could be due to there being more adult data points. We attempted to replicate the results of what vocalisation acoustics predict whether a response will be received, but found that with the human-labelled data, no effects were statistically significant, which could be due to the smaller number of data points (Table S13). We hope that future work will attempt to validate the WR vs. WOR and the age results presented here using a larger and more fully annotated human-labelled dataset.

**Table 3:** Inter-rater reliability measures for human-labelled data. Inter-rater reliability for each human listener with respect to the corresponding LENA labels are presented.

Listener id	Child id	Child age	Simple percent agreement	Cohen’s Kappa
L1	274	82 days	78.51	0.65
L1	340	183 days	85.61	0.78
L2	530	95 days	71.38	0.53
L3	340	183 days	85.70	0.78

All values reported have been rounded to two decimal points wherever possible.

## Discussion

This study set out to examine infant and caregiver vocal behaviour from a foraging perspective. We focused on step size distributions, which provide one approach to characterising an individual’s exploration (and exploitation) processes. They have proved useful in understanding spatial foraging, for example to measure effects of resource scarcity on foraging strategies, as well for studying some non-spatial cognitive functions in humans^43^.

First we tested for correlations between inter-vocalisation time and inter-vocalisation distance in acoustic space. Distance and flight or walk time are naturally correlated in spatial foraging; longer distances take longer to traverse than shorter distances. Prior research on memory foraging has found a correlation between inter-response-intervals and semantic distance^42,43^, and this finding has informed our understanding of the organisation and access of semantic memory. In the present study, we found that correlations between inter-vocalisation intervals and acoustic distance between vocalisations were weak (but very statistically reliable). One possible explanation for the finding that the correlation was weak is that our acoustic space did not provide a great representation of infant and adult vocalisation features. Our 2D acoustic space took into account only the mean pitch and amplitude of each utterance. It ignored all other temporal and spectral features, some of which are key to identifying major types of vocalisations such as canonical (syllabic) infant babble. On the other hand, there is good reason to believe that mean pitch and amplitude are key features that both adults and infants vary in their own vocalisations and attend to in each others’^28,30,31,39^. Errors in labelling the onsets and offsets of vocalisations and in the measurement of pitch could also have played a role in reducing the correlation between inter-vocalisation interval and distance in acoustic space. It is also possible that changing vocalisation acoustics can be swiftly modified more easily than walking or flying between distant physical locations and than traversing distant locations in semantic memory. With more accurate labelling and a more comprehensive acoustic space validated by listener judgments, we may either find that time-space correlations remain weak or that they are revealed to be as strong as those found in memory and spatial foraging. Either way, the results will be highly informative about infant and adult vocal exploration, so such future work will be quite worthwhile.

Inspired by the animal and memory foraging literature, we used AIC to determine whether exponential, normal, lognormal, or Pareto distributions best fit the inter-vocalisation step-size distributions for a given recording (and speaker type and response context). We found that step sizes in both time and space were largely lognormally distributed. This suggests that vocal search trajectories are clustered, a feature shared with spatial and memory foraging. On the other hand, the fact that step size distributions were not pareto (the exception being adult inter-vocalisation intervals split according to whether a response was just received) suggests that vocal foraging may not be a Lévy process, as has been found for those other domains. That said, future advances in vocalisation labelling and measurement will be important to confirm or modify this conclusion. It is also worth noting that we found that when acoustic dimensions were analysed individually, step size distributions were exponentially distributed. This is unsurprising since computing Euclidean distances on data points distributed exponentially in two orthogonal dimensions produces a probability density that is not exponential. However, the finding highlights that for exploration in more abstract, potentially high-dimensional spaces representing acoustic features (or semantic, visual, or other cognitive features), the distribution type may be less informative than comparisons of how distribution features co-vary with performance or other variables of interest.

Thus, perhaps more important than determining whether or not behaviour generally shows power law scaling is determining whether behaviour dynamics change, qualitatively and/or quantitatively, as a function of other variables of interest, such as infant maturity or resource availability. Inspired by previous studies of foraging behaviour we pursued a distribution fitting and parameter comparison approach to test for differences in vocal foraging dynamics as a function of age and recent resource acquisition, specifically recent receipt of a vocal response. The distribution fits provided a holistic perspective in the sense that they provide a complete characterisation of the shape of the step size or inter-event-interval distributions. On the other hand, we found the parameters of the fits somewhat challenging to interpret, especially in the case of lognormally distributed data, and thus we found that analysing median and 90^th^ percentile values provided a concrete and interpretable way of characterising the distributions. In many cases the two approaches provided converging, complementary evidence for how step size distributions related to response receipt and infant age.

Although we did not have a specific hypothesis regarding how infant age would relate to exploration, we did find intriguing differences in both infant and adult vocal foraging dynamics as a function of age. In particular, we found that as infants got older (and generally more skilled at vocalising) they explored with bigger steps in pitch but smaller steps in amplitude. We also found that with infant age, adult vocalisations to which infants were exposed exhibited longer times between adult vocalisations as well as bigger vocalisation-to-vocalisation differences in the adult pitch and amplitude (and the 2D acoustic space based on these two acoustic features). These findings seem at least in some cases to be related to changes in exploration dynamics rather than simply tracking age-related changes in amplitude and pitch ranges (Tables S2, S3 and Fig. S1).

A possible explanation for the infant finding is that over the course of the first year and a half of life infants become increasingly skilled at varying the pitch of their vocalisations and produce more active variation along this dimension either for their own interest or to elicit the interest of their caregivers^2^. A possible explanation for the adult finding is that as infants become more skilled communicators and more cognitively capable and socially aware in general, adults systematically vary their vocalisations in order to stimulate infants^31,34,53^. Another possible explanation might be that as infants get older they begin to spend more time physically distant from their caregivers. This could result in the adult vocalisations infants can clearly hear to be somewhat less frequent^54^, therefore having longer inter-vocalisation intervals and possibly as a result, correspondingly larger acoustic step sizes. Or it may be that as infants get older, the accuracy of the automatic labelling and measurement increases, revealing greater variation in the acoustic measures. Again, further exploration and development of labelling and acoustic measurement methods will be helpful in clarifying and better understanding these patterns.

As for social effects of receiving a vocal response on vocal foraging dynamics, we hypothesised that vocal responses from adults would function as rewards (among the resources being foraged for) for infants and vice versa. We expected that when a vocalisation was followed by a vocalisation from another speaker type, the speaker’s next vocalisation would occur more quickly and would be more similar to the previous vocalisation than when the preceding vocalisation was not followed by a vocal response.

Inter-vocalisation intervals supported foraging for social responses hypothesis: both infants and adults had shorter inter-vocalisation intervals (as indicated by median values and/or fitted step size distribution parameters) when the first vocalisation in a pair was followed by a response than when it was not. This finding fits well with the body of research on naturalistic infant-caregiver vocal interaction based on human-labelled utterance onsets and offsets – it has been found that across cultures infant speech-related vocalisations show bidirectional first order contingencies on each others’ occurrence at various ages throughout the first year of life and beyond^37–39^. This agreement between our data and prior work provides additional validation for our new approach focusing on step size distributions. It also provides converging evidence for bidirectional coupling between infant and adult vocalisation processes, and it supports our initial social foraging hypothesis. It should be noted however, that concerns about first order measures’ inability to resolve causal pathways (at least when based on non-experimental observations) apply as much to the present study as they do to the prior body of research^39^.

As for social effects on step sizes in acoustic space, we did not see a pattern of statistically significant effects that straightforwardly corresponded to our hypothesis in terms of paths being shorter following a response—the only statistically significant effects of response on infant acoustic path length were a tendency for pitch steps to be *larger* following response and a tendency for steps in 2-D space to have right-shifted distribution peaks and lighter tails corresponding to a smaller lognormal *σ* parameter. Moreover, the effects of infant responses on adult path lengths were also in the opposite direction from what we expected when considering the 2D acoustic space. The finding that adult acoustic step sizes were generally larger following an infant response could reflect the increased variability associated with infant-directed speech. However, since the effect was only apparent in the amplitude dimension and since amplitude is from the infant’s perspective and not the adults’, the results are perhaps more likely reflecting a tendency for adults to get closer to infants following an infant response. There was one statistically significant effect consistent with our initial hypothesis relating social response to reduced acoustic step size: adult steps in the pitch dimension tended to be shorter following an infant response.

One possible explanation for the null and unexpected results could be that our acoustic space is not comprehensive enough to capture enough of the key features that infants and adults repeat after getting a response^5,31–33,55,56^. Another possibility is that our definition of response receipt was not ideal, not taking into account enough temporal information and not taking into account any features of the responses, such as whether they were positive, who or what the vocalisation was directed toward, what their acoustic properties were, etc. Too much noise in the automatic labelling and the acoustic measurement could also have limited our ability to detect effects. Perhaps longer timescales where behaviour is not viewed at the utterance level but at the level of groups of utterances would also be good to consider (we did do some preliminary area-restricted search inspired plotting that looked at longer series of events, reported in Supplementary Figs. S6, S8, S11 and S13). And of course it is possible that infants really are minimally affected by adult responses. We will be more confident drawing conclusions once future work taking into account other possible acoustic spaces and with better human validation for the labelling has been conducted. Nevertheless, we believe the present study provides a useful framework for asking such questions about social effects on vocal exploration dynamics, and our initial results regarding the effects of infant responses on adult pitch and amplitude step sizes are intriguing.

Finally, we found that certain acoustic features of infant and adult vocalisations predicted whether a response would follow. In particular, we found that adult vocalisations that were higher in amplitude and higher in pitch were more likely to be followed by infant vocalisations. This supports prior literature indicating that acoustically modified infant-directed speech, which tends to have higher pitch together with greater frequency modulation, is more salient and appealing to infants^31,39,53,57,58^. It also supports our intuition that higher amplitude sounds are more salient to infants. Infant vocalisations were more likely to be followed by an adult vocalisation when they were lower in pitch and higher in amplitude. The higher amplitude finding can perhaps be explained by the greater salience of higher amplitude sounds, although to the extent that greater amplitude is associated with more mature infant vocalisations, that could also be playing a role. The fact that lower pitch infant vocalisations were more likely to be followed by an adult response might also be explained by those vocalisations sounding more adult-like and perhaps more speech-like. Similarly, we found that vocalisations that are further apart in time and pitch are less likely to receive both infant and adult responses, while vocalisations that are further apart in amplitude are more likely to receive both infant and adult responses. One possible explanation for the time step finding is that closely spaced vocalisation events are more noteworthy to the responder than those that are not. It could also be that events are temporally closer when infants and adults are interacting with each other and hence, responses are more likely during these periods of interaction. The findings about step sizes in pitch and amplitude are harder to interpret since these step sizes were non-directional. Repeating these tests with directional step sizes may be more informative. One way to think of larger amplitude steps being more likely to receive a response is that these could correspond to shifts to louder, and hence more noticeable, vocalisations. It will be interesting for future research to explore the underlying reasons for these effects as well as to identify other acoustic predictors of responses and to link these more directly with infant and adult foraging behaviour. For example, it may be that infant vocalisation acoustics which predict adult responses become targets for the infant’s vocal exploration even in the absence of an interactive adult caregiver, during times of more solitary vocal play. Our findings on vocalisation acoustics patterns predicting a response are especially interesting when juxtaposed with our findings that adult amplitude steps increase after receiving an infant response, while adult pitch steps decrease following an infant response. For example, it could be that adults repeat similar vocalisation patterns that yielded an infant response in an effort to elicit more responses. On the other hand, a tendency for LENA’s automatic labelling software to systematically mislabel infant vocalisations as adult speech and vice versa could have biased the results on how pitch predicts response in the exact directions observed here. Future validation using a more comprehensively human-labelled dataset is necessary.

For nearly all of our analyses, we had concerns about that automatic speaker labelling inaccuracies could have affected our results. In particular, if voice identification accuracy varies as a function of age, then differences in vocal foraging as a function of age could potentially reflect automatic labelling artefacts. Similarly, tendencies for the LENA system to misidentify adult vocalisations as infant vocalisations and vice versa are a potential confound for our results suggesting a relationship between response receipt and inter-vocalisation step sizes. Of all the results presented, the two we think are least likely to be artefacts of mislabelling are the finding that acoustic step size and inter-vocalisation interval are correlated and the finding that inter-vocalisation intervals are smaller following a response. One reason we are more confident in these results is because they appear to replicate prior findings obtained on different datasets using manually labelled data. But even in these cases, artefacts cannot be ruled out. Because of this general concern, we attempted to obtain human re-labellings of full day-long recordings, in order to see if we could use these to validate our results. Ultimately, we were not able to obtain enough of these re-labellings to have a large enough dataset that we could have the power and representativeness to establish whether or not our results would have held using human-labelled data. Unfortunately, this concern is one that holds for much of the research that has been published to date using the LENA system, and fortunately, it is one that researchers are increasingly attentive to and is an active topic of research^59^. The results reported (except perhaps for those exceptions mentioned above) should undergo a more extensive and rigorous validation using human listeners’ labels, and can also be compared to results obtained using alternative labelling methods just now becoming available^60,61^. It is also worth noting that an advantage of automatic labelling is that in principle it should make applying the same standard method to novel datasets both more feasible and more efficient. Moreover, although human listener judgments remain the gold standard, differences between human annotators and annotation protocols, and between analysis approaches and parameters, can also lead to challenges in interpreting results of analyses. We are optimistic about the value of the methodological approach described in the current paper, outlining a specific set of procedures for viewing infant and adult vocal communication as foraging processes with initial results suggesting that exploration patterns vary with age and that search patterns are influenced by social responses.

## Methods

Audio recordings for this study were obtained using the LENA™ Pro system, which consists of a small single-channel audio recorder and cloth vests each with a chest pocket into which the recorder can be placed. Caregivers were instructed to turn on the recorders at the beginning of the day, place them in the vest pockets, and then put the vests on their infants. The recorders can capture up to 16 hours of audio, including sounds made and heard by the infant.

Recordings included in the present study were obtained as part of two separate data collection efforts. The first involved two infant participants, one learning English and Spanish and the other learning German and English, who were recruited via acquaintance with one of the authors (ASW) and who began the study at 1–2 months of age and were recorded approximately twice per week until 11–13 months. Recordings were made on days that were convenient for the infants’ families, and the infants’ parents were instructed to turn on the recorder when the infant woke up in the morning and to turn it off when the infant was put to sleep for the evening. The recorder could be paused as needed for privacy purposes.

The second effort is ongoing and involved 15 participants from the Merced, CA region. Seven children were learning only English; four children were learning both English and Spanish with one of these children having Spanish as the primary language; one was learning English, Spanish, and Sahaptin; one was learning English and German; one was learning English with a small amount of French input; and one was learning English together with another language not specified by the caregiver. Infants were recruited via word of mouth, flyers, and in-person recruitment at a local hospital and community events. The infants in this study were scheduled to be recorded for at least 10 hours during a single day at 3, 6, 9, and 18 months of age. Many of the later recordings had not yet been collected by the time data were prepared for analysis for the present paper. Caregivers were asked to record on days when most of the infant’s immediate family members were home and when outings and special events, especially involving a lot of socialising, would be minimal. In most cases, recordings were made on typical weekend days. Caregivers were instructed to turn on the recorder in the morning, no later than 8 am and to turn off the recorder in the evening, no earlier than 7 pm. Caregivers were told that they could pause the recorders for privacy purposes, but that pause time should not exceed one hour total over the course of the target recording period. Parents were able to have the researchers delete sections of the recording when private events took place and they had not been able to pause the recorder in advance. Caregivers also filled out various questionnaires some of which were to be completed on the day of recording, and were provided in most cases with cash compensation for their time assisting with the study. Since caregivers in both data collection efforts were instructed to complete the recording on a single day, we use the infant’s age in days on the day of recording for infant age. However, we have not controlled for possible recordings split over multiple days due to caregiver error, and these recordings, if they exist, have infant age as the infant’s age in days on the day of the first subrecording.

Both datasets were collected in accordance with relevant guidelines and regulations. Informed consent was obtained from the infant participants’ legal guardians, and data collection protocols were approved by the University of Memphis Institutional Review Board (dataset 1) and by the University of California, Merced Institutional Review Board (dataset 2).

Once recordings were completed, caregivers returned the audio recorders to the researchers, and the audio recordings were uploaded to and processed by the LENA Pro software. The software automatically segments each audio recording into a mutually exclusive and exhaustive set of sound source segments, using a speech recognition algorithm involving a combination of Gaussian mixture and hidden Markov models. The algorithm was previously trained on human-labelled LENA recordings collected by the LENA Research Foundation and is dependent on the infant’s age in months. All subsequent analyses focused exclusively on segments labelled “CHN” (clear vocalisations by the infant wearing the recorder^62^) containing at least one “Utt” (a speech-related vocalisation, as opposed to cry, laugh, or vegetative vocalisations), called “CHNSP” in our analyses; “FAN” (clear adult female vocalisations); and “MAN” (clear adult male vocalisations). Note that adult vocalisations collectively refer to FAN and MAN labels, i.e., adult vocalisations are not necessarily from a single adult. A study of the system’s sensitivity in detecting various sound sources, conducted by the LENA Research Foundation^62^, reported that 82% of portions identified by human transcribers as adults vocalising near the infant were correctly identified by the system as FAN or MAN, and 2% were mislabelled by the system as infant. For portions identified by human transcribers as infant vocalisations, 76% were correctly labelled as such by the system and 7% were mislabelled as adult. Specificity data were not included in the report.

For three of the day-long recordings, research assistants used custom software (source code available at https://github.com/tim-shea/ivfcr-server) to listen to the audio clips that were labelled by the LENA Pro software as either CHN, CXN (near-sounding vocalisations by an infant other than the one wearing the recorder), FAN, or MAN. The research assistants then indicated which speaker types they actually heard during those clips, entering multiple labels for a segment if multiple speaker types were heard. One of the three recordings was labelled by two different people, allowing for comparison of results across raters (one recording, of a 6 month old, was re-labelled by listeners 1 and 3, a recording of a different participant at 3 months was re-labelled by listener 2, and a recording of yet another participant at 3 months was labelled by listener 1). Vocalisations were listened to in the order in which they occurred and were mostly labelled in that same order, except when listeners opted to skip the sounds and return to them later. (Note that a small percentage of the segments identified by LENA did not receive human listener labels due to some issues with the software not returning users to vocalisations they had opted to skip and return to later. In all step-size analyses, this would have resulted in a small set of steps that are not between temporally sequential infant-to-infant and adult-to-adult step sizes in the data re-labelled by human listeners.) This allowed us to obtain an independent measure of the accuracy of the labels as well as to run analyses on recordings with more accurate labels (human listeners, while more idiosyncratic than the automatic labelling software and not perfectly reliable in their judgments, are generally accepted to be much more accurate than the LENA Pro software) so that results could be compared to LENA-labelled recordings to help identify whether inaccuracies in the automatic labelling could have biased our results. For all validation analyses using data re-labelled by human listeners, we used vocalisations with MAN, FAN, or CHN labels, and without multiple speaker labels only. Further, since all our analyses on LENA-labelled data were carried out on infant vocalisation data with CHNSP labels only, we filtered the CHN labels by human listeners (which comprised of both CHNSP and non-CHNSP vocalisations) using the CHNSP labels by LENA before proceeding with any analyses. MAN and FAN segments that were labelled by humans as CHN were kept and were presumed to be predominantly CHNSP. We are less concerned with missed vocalisation instances (which due to the nature of the human labelling task were likely quite common) than we are with instances where an adult vocalisation is mistaken for a vocalisation by the infant wearing the recorder and vice versa, as such instances would be more problematic for the research questions posed here.

The automatically obtained vocaliser label data were then used to segment each recording’s audio into individual WAV files for each CHN (although only CHNSP were used in our analyses) and AN (FAN or MAN) utterance. A pitch contour for each utterance was then automatically obtained using Praat^63^, using the auto-correlation method with a minimum pitch of 75 Hz and a maximum of 1000 Hz for both infant and adult vocalisations. The wide range of possible pitch values was used because it is known that infants and adults interacting with infants tend to vocalise with fundamental frequency that often exceeds the range, on both the low and high ends, of fundamental frequency in typical adult-directed adult speech^55,56^. Average intensity (a measure of amplitude) was also automatically obtained in Praat for each utterance. In some instances, Praat did not detect a clear pitch for any portion of the utterance; these utterances were subsequently excluded from all analyses. For each utterance, we then obtained the log of the mean pitch (fundamental frequency) in Hz and the mean intensity in dB. Both pitch and amplitude were then converted to z-scores with all infant (CHNSP) and adult (FAN, MAN) vocalisations included in the dataset for standardisation. The standardised log mean pitch and mean intensity were then used to position each utterance in a two-dimensional pitch-amplitude space.

We also used the time stamps of the automatically obtained vocaliser labels to determine whether each infant vocalisation was followed by an adult vocalisation within 1 s following the offset of the infant vocalisation, in which case we (operationally) say that the infant vocalisation received a response. We used the same to determine whether each adult vocalisation was followed within 1 s by an infant speech-related (CHNSP) vocalisation, in which case we say that the adult vocalisation received a response. In cases where two infant vocalisations (CHNSP or non-CHNSP) occurred with less than 1 s separation intervening and no adult vocalisation occupied the intervening time, we marked adult response to the first infant vocalisation as ‘not applicable’, and the same was done for two adult vocalisations occurring with less than 1 s separating intervening and no infant vocalisation occupying the intervening time^21^. For human re-labelled data, a similar approach but using labels as assigned by human listeners was used to determine whether a vocalisation received a response or not, or whether a response was ‘not applicable’ (there were some minor differences in the way ‘not applicable’ was defined; see analysis code for details). We determined acoustic measures (standardised mean log pitch and standardised amplitude) for human-labelled data by matching start times of vocalisations to LENA-labelled data and using the associated acoustic measures.

Linear mixed effects analyses were run predicting day-level recording step size distribution features. As fixed effects, we entered infant age, the reception of adult/infant response, and sample size (the number of CHNSP, MAN, and FAN vocal events in the recording) into the model. Analyses including an age*response interaction term always returned null effects for the interaction; thus we excluded the interaction term from all analyses reported here. We performed separate analysis with and without the interaction term and the sample size term (see Supplementary). Participant ID was always included as a random effect. For these analyses, any pauses of the recorder were filtered out by removing step sizes corresponding to the step from the end of one recording to the beginning of the next – thus, all data analysed are day-level recordings. All step sizes were also non-directional, i.e., we used absolute values of differences in pitch and amplitude for all analyses.

Finally, we ran logistic mixed effects regressions to determine whether certain vocalisations/vocalisation patterns were more likely to receive responses than others. We used a binary response variable (1 for response received, and 0 for no response) as the dependent variable. To test the relationship between the amplitude and pitch of vocalisations and the probability of a vocalisation receiving a response, we entered infant age, z-scored log pitch of the utterance, and z-scored amplitude of the utterance into the model as fixed effects. To test the relationship between step sizes in pitch, amplitude, and time leading to a vocalisation, and the probability of the vocalisation receiving a response, we added step sizes in z-scored log pitch, z-scored amplitude, and time, from vocalisation $i-1$ to vocalisation *i* (where vocalisation *i* is the vocalisation of interest) as fixed effects, in addition to z-scored log pitch of the utterance and z-scored amplitude of the utterance, and infant age. Participant ID was treated as a random effect.

Following pre-processing, MATLAB (R2019a) was used for all analyses except for the mixed effects regressions, for which R (version 3.5.2 – “Eggshell Igloo”), and packages lme4 and lmerTest (Bates, Maechler & Bolker, 2012) were used.

## Supplementary information

Supplementary Information

## Data Availability

Analysis code and de-identified pre-processed data used in the present paper are available on at https://github.com/AnneSWarlaumont/infant-vocal-foraging and https://osf.io/8ern6/, respectively. In addition, https://osf.io/8ern6/ also has all intermediate files used in analyses, as well as all results reported in this paper and Supplementary Information. The raw audio data and LENA labels for participants who gave permission for us to share their data with other researchers are available within the Warlaumont corpus in HomeBank^64,65^.
