# Supplementary material for: Exploratory dynamics of vocal foraging during infant-caregiver communication
Source: Sci Rep. 2020 Jun 26;10:10469. doi: 10.1038/s41598-020-66778-0 (PMC7319970; doi:10.1038/s41598-020-66778-0)
Supplement: Supplementary file 1 — Supplementary Information [file 41598_2020_66778_MOESM1_ESM.pdf]

Supplementary Information: Exploratory dynamics of vocal foraging during infant-caregiver communication

Ritwika VPS[1*], Gina M. Pretzer[2], Sara Mendoza[2], Christopher Shedd[1], Christopher T. Kello[2], Ajay Gopinathan[1], Anne S. Warlaumont[3**]

[1]University of California, Merced, Department of Physics, Merced, CA 94343, USA; [2]University of California, Merced, Cognitive and Information Sciences, Merced, CA 95343, USA; [3]University of California, Los Angeles, Department of Communication, Los Angeles, CA 90095, USA

[*]ritwika@ucmerced.edu [**]warlaumont@ucla.edu

**Ch**	Infant vocalisation	**Ad**	Adult vocalisation
**WR**	With response (for steps following vocalisations that received responses)	**WOR**	Without response (for steps following vocalisations that did not receive responses)
**LENA or L**	Labelled by LENA software	**HUM or H**	Labelled by human listeners
**(f)**	AIC best fit curve	**(d)**	Raw data-based curve
**s.z.**	Step size	**std. dev.**	Standard Deviation

Table S1: **List of abbreviations in figure legends and axis labels**

S1. Infant and adult acoustics* as a function of infant age

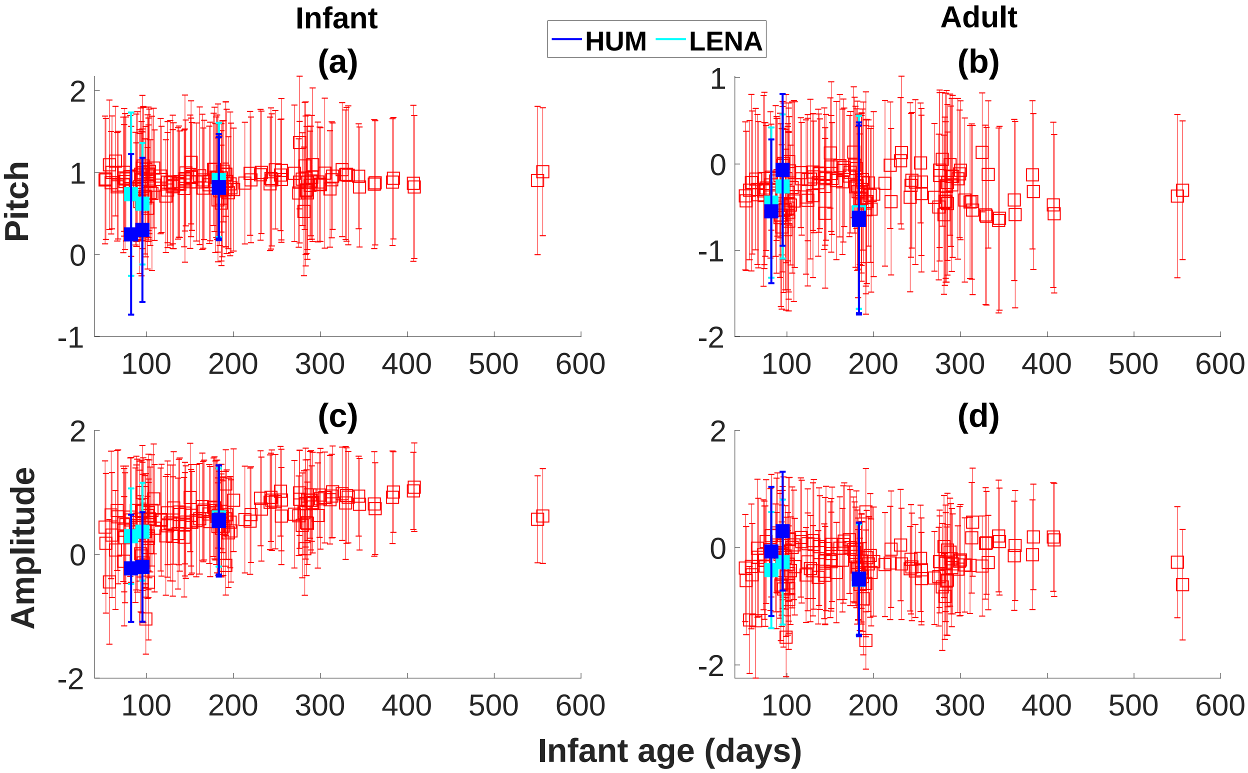

Figure S1: **Infant and adult vocalisation acoustics as a function of infant age.** Mean pitch (standardised across all infant and adult vocalisations combined) as a function of infant age for infants (panel a) and adults (panel b). Red squares are for the complete dataset, automatically (LENA) labelled. Each data point corresponds to one day-long recording. Error bars represent the standard deviation**. The dark blue points (HUM in the legend) represent the same information as computed from data labelled by human listeners, while the cyan points (LENA in the legend) are the values obtained from the corresponding data as labelled by the LENA system. Note that data from infant 340 at 183 days was re-labelled by two human listeners and results from both listeners are represented. (c, d) Mean standardised amplitude of vocalisations as a function of infant age for infants and adults, respectively.

*In all our analyses, acoustic measures – amplitude and log pitch – are unitless since they have been standardised.

**We computed the unbiased sample standard deviation, dividing by N-1, where N is the number of samples used in computing the standard deviation. This is in contrast to the population standard deviation, which divides by N.

Measure	Age effect	Measure	Age effect
Infant mean pitch	0.11 (p = 0.13)	Infant pitch standard deviation	**0.21 (p = 0.01)**
Infant mean amplitude	**0.50 (p < 0.001)**	Infant amplitude standard deviation	**-0.61 (p < 0.001)**
Adult mean pitch	-0.12 (p = 0.13)	Adult pitch standard deviation	**0.31 (p < 0.001)**
Adult mean amplitude	0.001 (p = 0.99)	Adult amplitude standard devitation	**-0.35 (p < 0.001)**

Table S2: **Vocalisation acoustics as a function of infant age**. βs are shown with p-values in brackets. Both were obtained from linear mixed effects models with participant ID as a random effect and infant age as fixed effect. Statistically significant results (at a significance level of 0.05) are in bold. All values reported have been rounded to two decimal points wherever possible.

Measure	Response	Infant age	Response	Infant age	Infant age-response interaction
Infant mean pitch	**-0.91 (p < 0.001)**	**0.14 (p = 0.003)**	**-0.91 (p < 0.001)**	0.09 (p = 0.19)	0.11 (p = 0.22)
Infant pitch std. dev.	-0.14 (p = 0.19)	**0.26 (p < 0.001)**	-0.14 (p = 0.19)	**0.22 (p = 0.01)**	0.08 (p = 0.44)
Infant mean amplitude	**0.31 (p < 0.001)**	**0.51 (p < 0.001)**	**0.31 (p < 0.001)**	**0.62 (p < 0.001)**	**-0.22 (p = 0.01)**
Infant amplitude std. dev.	**-0.25 (p = 0.01)**	**-0.54 (p < 0.001)**	**-0.25 (p = 0.01)**	**-0.65 (p < 0.001)**	**0.21 (p = 0.02)**
Adult mean pitch	**0.80 (p < 0.001)**	**-0.22 (p < 0.001)**	**0.8 (p < 0.001)**	**-0.14 (p = 0.04)**	-0.17 (p = 0.06)
Adult pitch std. dev.	**-0.56 (p < 0.001)**	**0.25 (p < 0.001)**	**-0.56 (p < 0.001)**	**0.25 (p < 0.001)**	-0.0002 (p = 1.00)
Adult mean amplitude	**1.02 (p < 0.001)**	-0.03 (p = 0.49)	**1.02 (p < 0.001)**	0.04 (p= 0.50)	-0.13 (p = 0.09)
Adult amplitude std. dev.	**-0.23 (p = 0.02)**	**-0.22 (p < 0.001)**	**-0.23 (p = 0.02)**	**-0.38 (p < 0.001)**	**0.31 (p = 0.001)**

Table S3: **Vocalisation acoustics as a function of whether the vocalisation was preceded by response and infant age, with optional response-infant age interaction**. βs are shown with p-values in brackets. Both were obtained from linear mixed effects models with participant ID as a random effect, and whether the preceding vocalisation received a response and infant age as fixed effects. Interaction between response and infant age was used as an optional fixed effect. Results from the model with the interaction term are given in columns 4, 5, and 6. Statistically significant results (at a significance level of 0.05) are in bold. All values reported have been rounded to two decimal points wherever possible.

S2. Steps in time are correlated with steps in acoustic space

Vocaliser	Time step	Infant age
Adult	**0.05 (p < 0.001)**	**0.06 (p < 0.001)**
Adult (WR)	**0.11 (p < 0.001)**	**0.07 (p < 0.001)**
Adult (WOR)	**0.05 (p < 0.001)**	**0.06 (p < 0.001)**
Infant	**0.07 (p < 0.001)**	0.01 (p = 0.06)
Infant (WR)	**0.09 (p < 0.001)**	**0.02 (p = 0.002)**
Infant (WOR)	**0.06 (p < 0.001)**	0.001 (p = 0.76)

Table S4: **Step sizes in 2D acoustic space as a function of steps in time**. βs are shown with p-values in brackets. Both were obtained from linear mixed effects models with participant ID as a random effect. Infant age and steps in time were used as fixed effects, with presence or absence of a response and infant age-response interaction as optional fixed effects, to test the effect of receiving a response and/or potential interactions between infant age and response received on how steps in 2D acoustic space correlate with steps in time. Statistically significant results (at a significance level of 0.05) are in bold. All values reported have been rounded to two decimal points wherever possible.

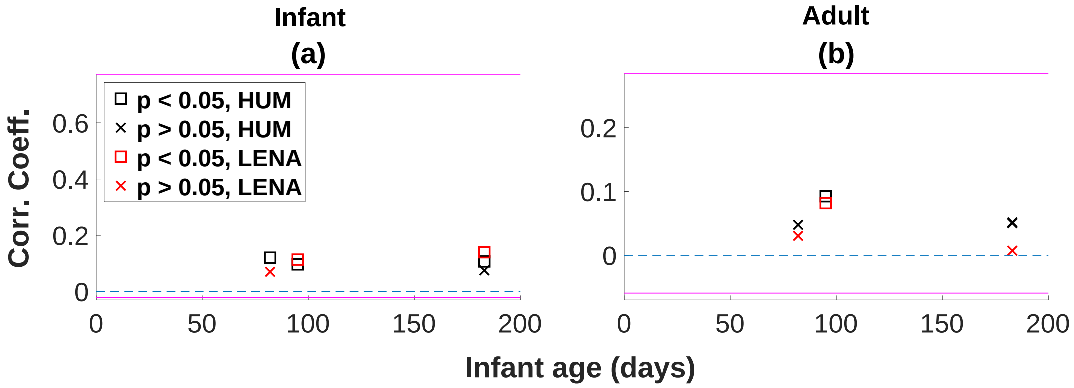

Figure S2: **Correlations between steps in acoustic space and steps in time validated using data labelled by human listeners.** In (a) and (b), squares represent significant correlations, while crosses represent correlations that were not significant (at a significance level of 0.05), for infant and adult vocalisations, respectively. Black points are obtained from data labelled by human listeners, while red points are from the same data labelled by the LENA software. Note that data from infant 340 at 183 days was re-labelled by two human listeners and results from both listeners are represented. The pink lines indicate the range of space-time correlation values obtained from all available data as labelled by LENA. The dashed blue line separates positive and negative correlation strengths. Note that these correlations were computed using MATLAB's corrcoef function.

S3. Step size probability density fits

For each set of step sizes – infants' pitch steps following an adult response, infants' pitch steps following no adult response, etc. – we used AIC (see `https://github.com/AnneSWarlaumont/infant-vocal-foraging/tree/master/Analyses/AIC_Theo_Rhodes_code` for details) to determine the best fit probability density distribution type and the curve parameters. The types of distributions considered were normal, lognormal, exponential and pareto distributions. For a given type of step, we determined what type of distribution best fit the majority of day-long recordings. We then analysed distribution parameters only from those recordings for which the best fitting distribution also belonged to that of the majority best fit type, for that set of step size distribution fits.

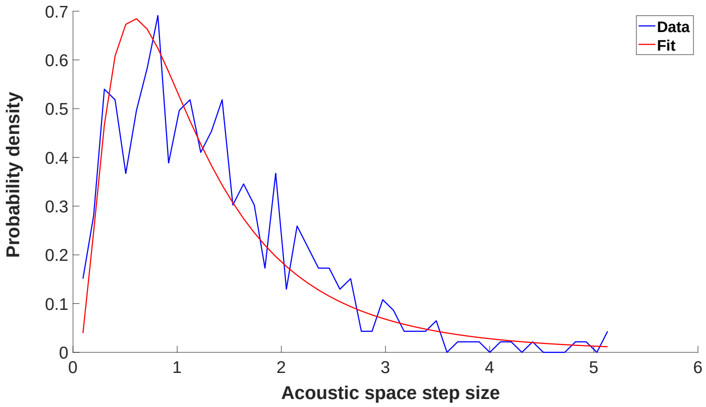

Figure S3: **Representative example of randomly selected data vs. AIC fit (Infant *mww*, age 75 days).** The figure shows the probability distribution of steps in acoustic space for infant vocalisations where the first infant vocalisation was not followed by an adult response. The distribution derived from the data is in blue and the AIC best fit (lognormal, in this case) is shown in red.

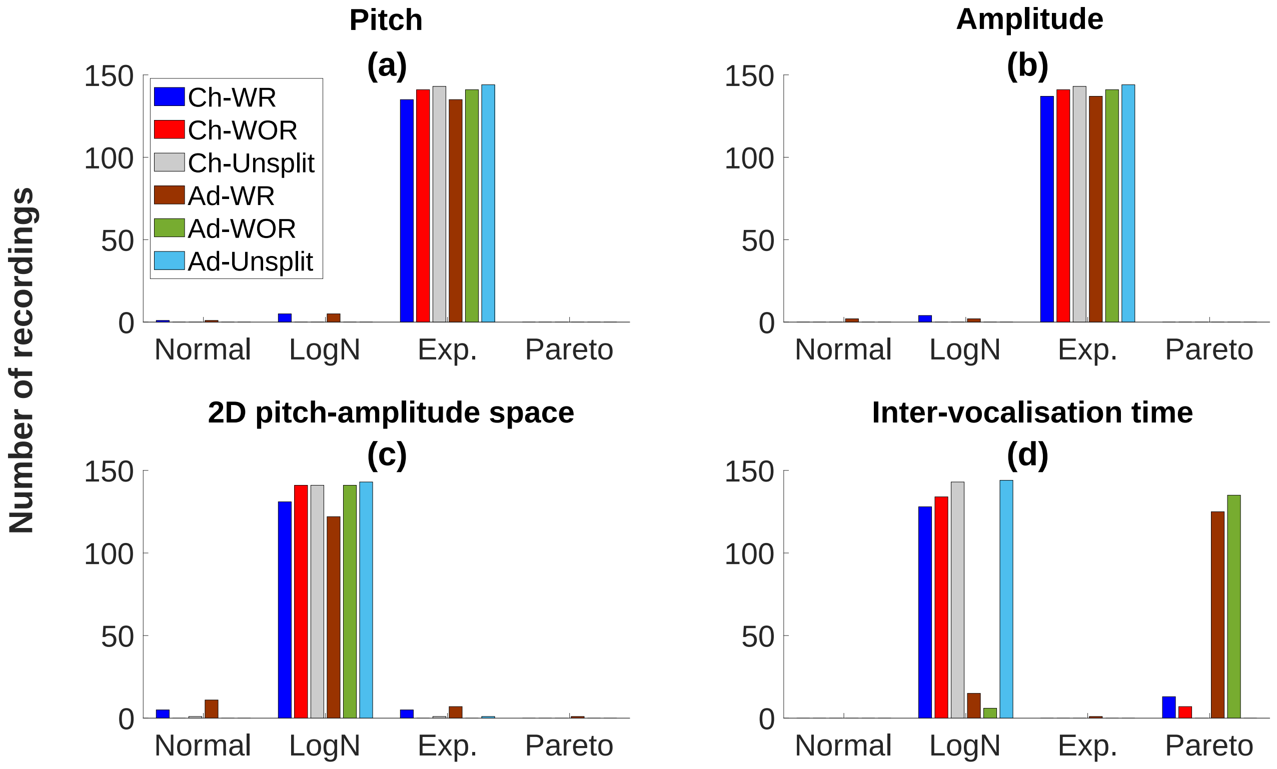

Figure S4: **Distribution of AIC best fits for step size probability distributions for various step types.** Probability distributions of step sizes along pitch (panel a) and amplitude (panel b) dimensions, respectively, are predominantly exponential, for both adults and infants. (c) Probability distributions of steps in two dimensional acoustic space are predominantly lognormal for both infants and adults. (d) Probability distributions of inter-vocalisation times are predominantly lognormal for infants, and pareto for adults with the exception that unsplit inter-vocalisation time distributions are largely lognormal for adults.

**Vocaliser**	**Step type**	**Mean R^2**	**Std. dev**	**Mean observations per distribution**	**Number of distributions**
Infant	Unsplit	0.92	0.10	1043.86	572
Infant	WOR	0.91	0.09	603.18	564
Infant	WR	0.78	0.20	184.83	564
Adult	Unsplit	0.94	0.08	3223.40	572
Adult	WOR	0.96	0.04	2031.54	564
Adult	WR	0.60	0.40	137.27	564

Table S5: **Goodness of AIC fits.** The means and standard deviations of the R^2 values of the AIC best fit for different step size distribution types are shown. All results are from data labelled by LENA. The step size distributions are organised by whether they were computed from data where the vocaliser was an infant or adult (column 1), and whether they are WOR, WR, or unsplit distributions (column 2). For mean and standard deviation for each step size distribution type for a category (eg. WR pitch step size distributions of adult vocalisations), see `https://osf.io/53amv/`. For a breakdown of the majority best fit for each distribution type, see Fig. S4. The fifth column contains the mean number of observations per distribution for that category while the sixth column has the total number of distributions that went into calculating the mean and standard deviation of R^2 values for that category. For example, the first row of the table gives the mean and standard deviation of R^2 values of all unsplit step size distributions (pitch, amplitude, 2d acoustic space, and time) where the vocaliser was an infant, regardless of best fit type. For this category, each distribution, on average, had 1043.86 observations, and 572 distributions were used to calculate the mean and standard deviation R^2 values. R^2 values typically fall between 0 to 1, with values closer to 1 indicating better fits. Note that one possible reason for lower R^2 values could be that some step types were less prevalent and therefore had fewer steps on which to fit the distribution (see fifth column of the table). All values reported have been rounded to two decimal points wherever possible. For a similar table for human-labelled data and the corresponding LENA-labelled subset, see section S4.

S3.1 Do amplitude step sizes vary with response and infant age?

		Median	90th percentile	Exponential parameter, λ	Median	90th percentile	Exponential parameter, λ
		**Infant vocalisations**			**Adult vocalisations**		
**With sample size**	Response	-0.13 (p = 0.25)	-0.18 (p = 0.09)	0.17 (p = 0.11)	**0.47 (p < 0.001)**	**0.37 (p < 0.001)**	**-0.43 (p < 0.001)**
	Infant age	-0.08 (p = 0.17)	**-0.24 (p < 0.001)**	**0.15 (p = 0.01)**	**0.23 (p < 0.001)**	**0.11 (p = 0.02)**	**-0.19 (p < 0.001)**
	Sample size	-0.13 (p = 0.12)	-0.12 (p = 0.13)	0.10 (p = 0.24)	0.06 (p = 0.37)	0.07 (p = 0.24)	0.01 (p = 0.92)
**W/o sample size**	Response	-0.13 (p = 0.25)	-0.18 (p = 0.09)	0.17 (p = 0.1)	**0.47 (p < 0.001)**	**0.37 (p < 0.001)**	**-0.43 (p < 0.001)**
	Infant age	-0.11 (p = 0.05)	**-0.27 (p < 0.001)**	**0.17 (p = 0.002)**	**0.23 (p < 0.001)**	**0.12 (p = 0.02)**	**-0.19 (p < 0.001)**

Table S6: **Steps in amplitude at the day-long recording level as a function of recent response, infant age, and sample size: results of statistical analyses**. βs are shown with p-values in brackets. Statistically significant results (at a significance level of 0.05) are in bold. Fixed effects are in rows and dependent variables are in columns. A separate linear mixed effects regression model was run for each dependent variable. Sample size was included to control for possible co-variation between sample size (number of vocalisation step events in the recording) and distribution fits, age, and response; including sample size did not affect which results were statistically significant or their sign. Infant ID was a random effect in all models. All values reported have been rounded to two decimal points wherever possible.

As shown in Table S6 and Figure S5, we observed a significant decrease with age in the 90th percentile value of infant amplitude step size. We also observed a significant increase with age in the λ parameter of the fitted exponential distributions. Both findings suggest that infants take shorter steps in amplitude as they get older, indicating more focused exploration. For adults' amplitude step sizes, we observed a significant increase in the median and 90th percentile values, as well as smaller λ for the exponential fits following infant response and with increasing infant age. These findings all point to larger steps in amplitude, and thus suggest broader adult exploration, both with infant response and with increasing infant age.

Finally, Figure S6 shows how, for infant and adult vocalisations from the entire dataset (all recordings at all ages combined), the median and 90th percentile values of amplitude steps change as a function of the number of vocalisation events by the speaker since a response was last received. Here, the step from the vocalisation that receives a response to the next vocalisation is designated as vocalisation 0, the following step is designated 1, and so on. This continues until the next response is received, at which point the count resets to 0. Thus, as shown in panels (c) and (d), there are fewer steps included in the analysis as the number of events since last response increases, and thus the estimates in panels (a) and (b) can be expected to be less stable as the number of vocalisations since last response increases. At the moment, we treat these visualisations as exploratory, leaving statistical analyses of such multi-event sequences for future work.

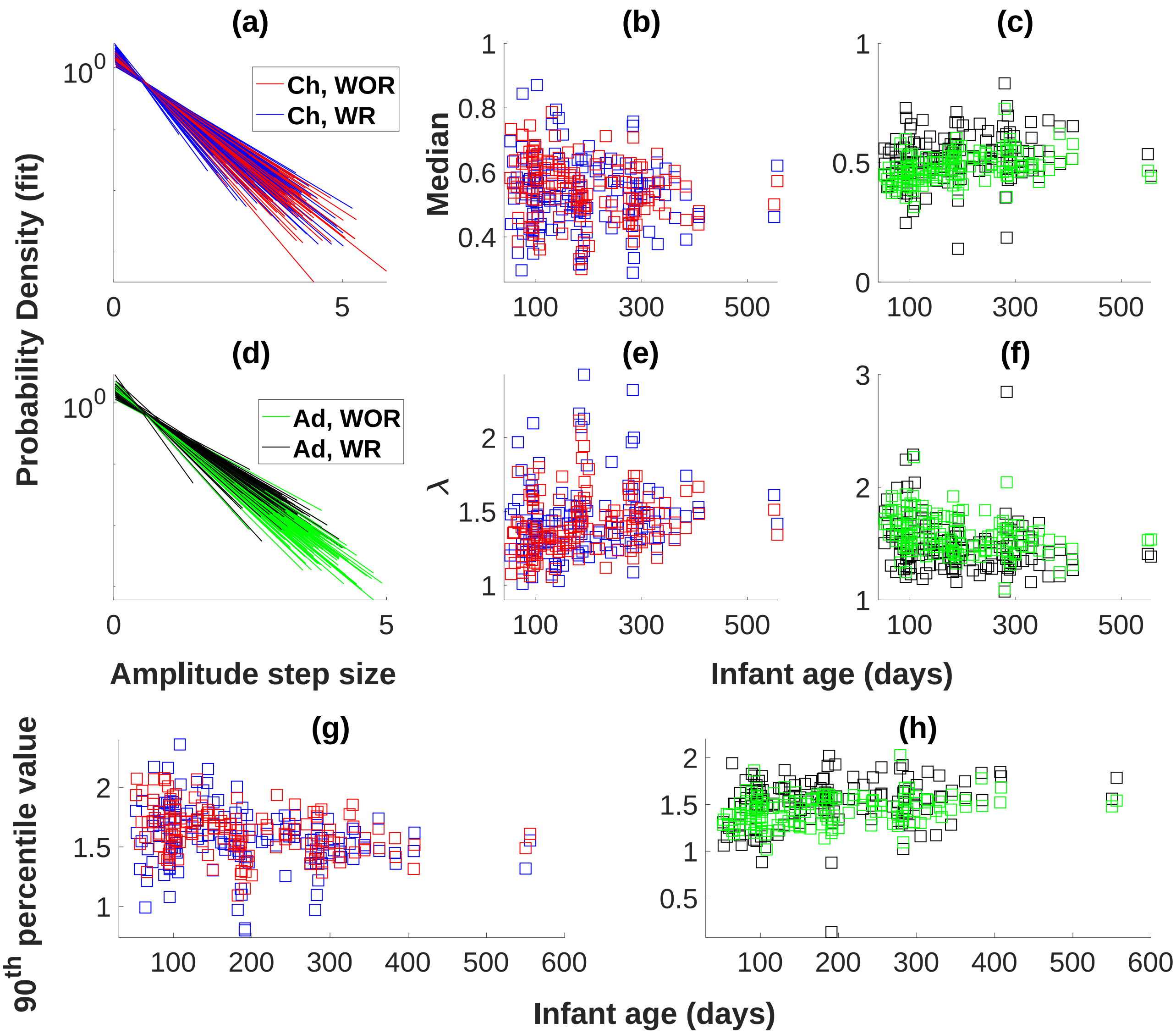

Figure S5: **Results of amplitude step size distribution analyses.** (a) Exponential probability distributions of infant inter-vocalisation changes ('step sizes') in amplitude following adult response (WR, blue) and not (WOR, red). (b) Median infant amplitude step size as a function of infant age, WR (blue) and WOR (red); (c) shows a similar plot for adults where WR is in black and WOR is in green. (d) Exponential distributions of amplitude steps for adults (WR, black; WOR, green). (e) Age-dependent change in the exponential paramater λ of the WR (blue) and WOR (red) exponential amplitude step size probability distributions for infants; (f) shows a similar plot for adults (WR, black; WOR, green). Note that only distributions that were determined to best fit to an exponential based on AIC are represented in (a), (d), (e), and (f). 90th percentiles of infant amplitude step size distributions plotted against infant age following adult response (WR, blue) and not (WOR, red); (h) shows a similar plot for adult amplitude step sizes (WR, black; WOR, green). Medians and 90th percentile values were computed based on the raw data prior to determining best fits using AIC.

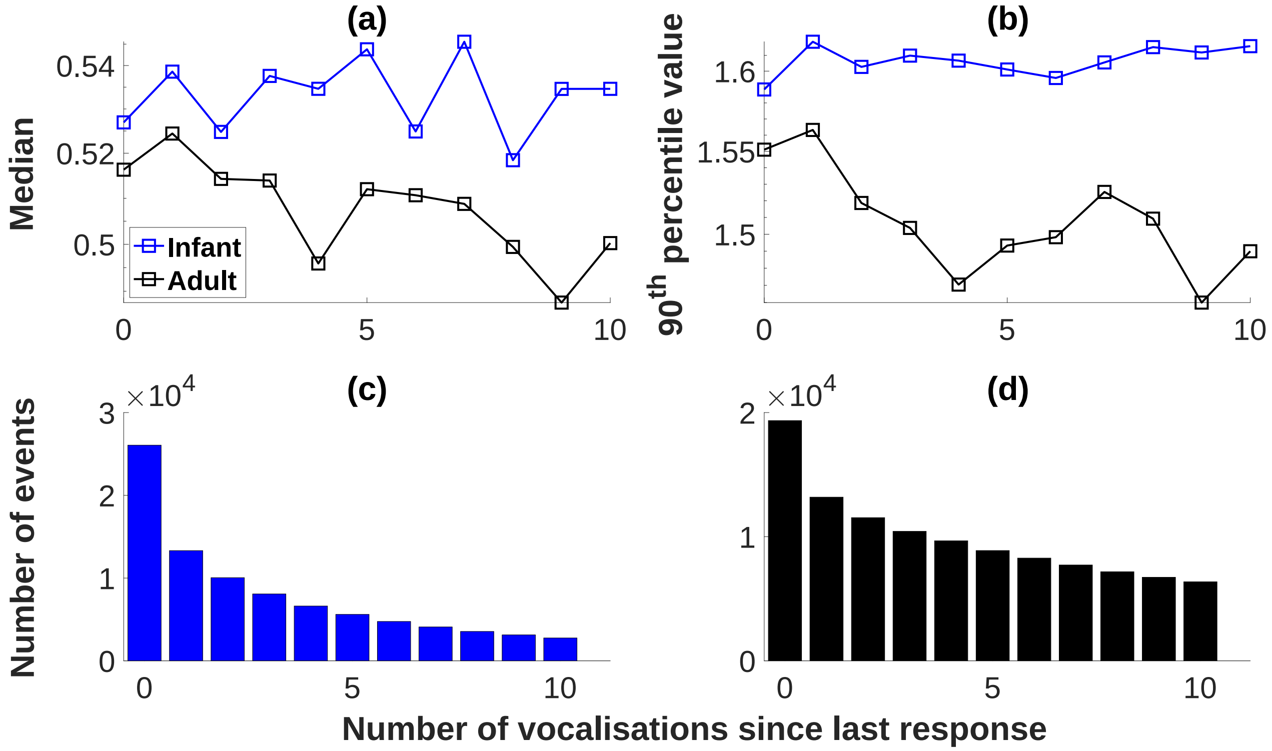

Figure S6: **Median and 90th percentile values for amplitude steps as a function of number of events since last response.** (a) Median amplitude step sizes for the entire dataset as a function of number of vocalisations since a response was last received. Here, the step from the vocalisation that received a response to the next vocalisation was assigned 0, the following step (assuming no response to the second vocalisation) was designated 1, and so on. This continued until the next response was received at which point the count resets to 0. The data for infants is in blue and that for adults is in black. (b) 90th percentile values of amplitude step sizes for the entire dataset, as a function of number of vocalisations since the last response was received (Infant, blue; adult, black). Number of vocalisation events as a function of number of vocalisations since the last response was received for infants (c), and adults (d). Note that plots were terminated after the 10th vocalisation step following the post-response step.

S3.2 Do pitch step sizes vary with response and infant age?

		Median	90th percentile	Exponential parameter, λ	Median	90th percentile	Exponential parameter, λ
		**Infant vocalisations**			**Adult vocalisations**		
**With sample size**	Response	**0.28 ($p = 0.01$)**	-0.01 ($p = 0.91$)	-0.11 ($p = 0.27$)	0.06 ($p = 0.50$)	**-0.19 ($p = 0.04$)**	**0.18 ($p = 0.03$)**
	Infant age	**0.25 ($p < 0.001$)**	**0.43 ($p < 0.001$)**	**-0.37 ($p < 0.001$)**	**0.31 ($p < 0.001$)**	**0.32 ($p < 0.001$)**	**-0.40 ($p < 0.001$)**
	Sample size	**-0.29 ($p < 0.001$)**	**-0.32 ($p < 0.001$)**	**0.35 ($p < 0.001$)**	0.04 ($p = 0.51$)	**0.13 ($p = 0.04$)**	-0.17 ($p = 0.01$)
**W/o sample size**	Response	**0.28 ($p = 0.01$)**	-0.01 ($p = 0.91$)	-0.10 ($p = 0.35$)	0.06 ($p = 0.50$)	**-0.19 ($p = 0.04$)**	**0.17 ($p = 0.04$)**
	Infant age	**0.19 ($p < 0.001$)**	**0.36 ($p < 0.001$)**	**-0.30 ($p < 0.001$)**	**0.31 ($p < 0.001$)**	**0.33 ($p < 0.001$)**	**-0.41 ($p < 0.001$)**

Table S7: **Steps in pitch at the day-long recording level as a function of recent response, infant age, and sample size: results of statistical analyses**. βs are shown with p-values in brackets. Statistically significant results (at a significance level of 0.05) are in bold. Fixed effects are in rows and dependent variables are in columns. A separate linear mixed effects regression model was run for each dependent variable. Sample size was included to control for possible co-variation between sample size (number of vocalisation step events in the recording) and distribution fits, age, and response; including sample size did not affect which results were statistically significant or their sign. Infant ID was a random effect in all models. All values reported have been rounded to two decimal points wherever possible.

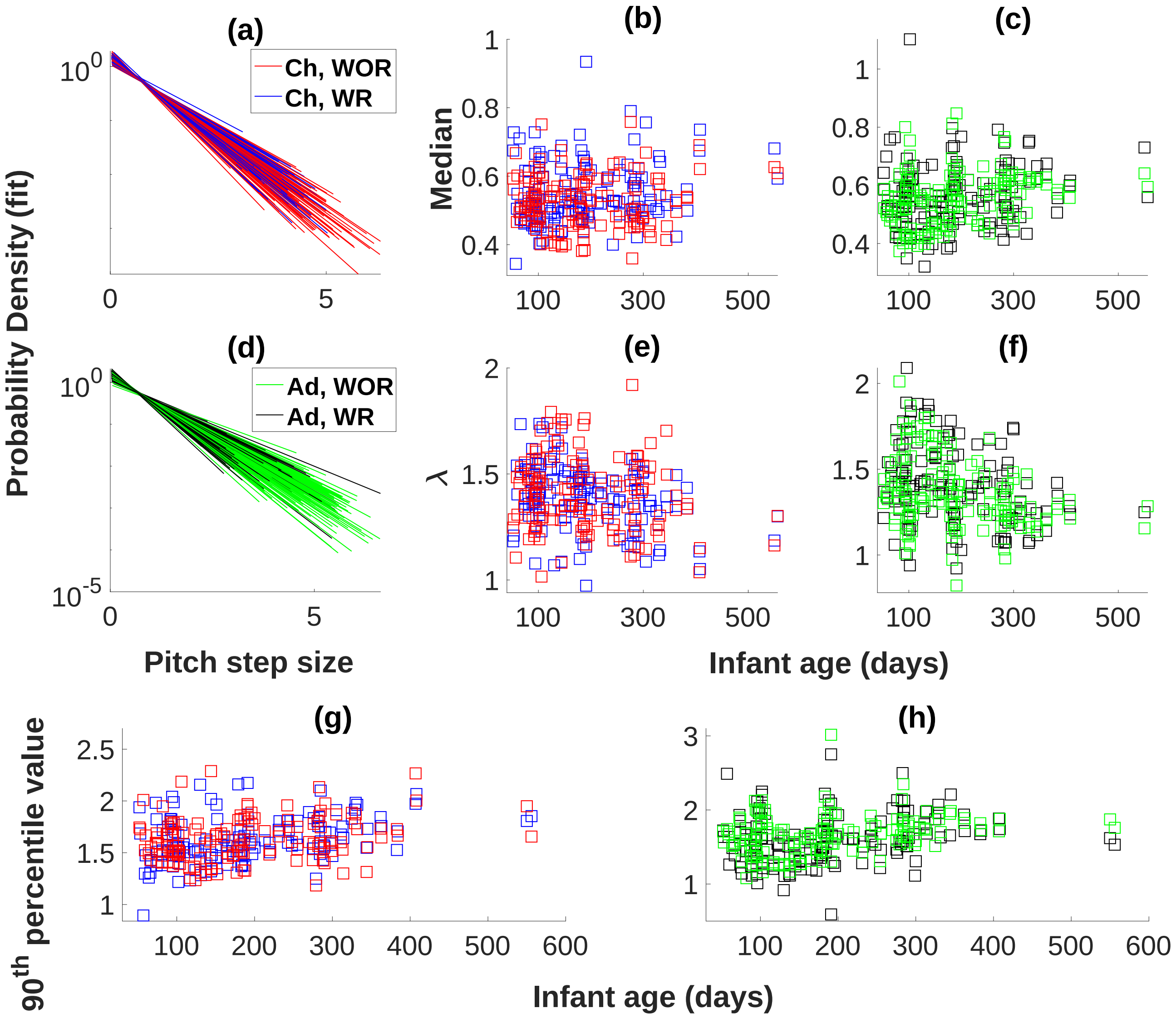

Figure S7: **Results of pitch step size distribution analyses.** (a) Exponential probability distributions of infant inter-vocalisation changes ('step sizes') in pitch following adult response (WR, blue) and not (WOR, red). (b) Median infant pitch step size as a function of infant age (WR in blue and WOR in red); (c) shows a similar plot for adults where WR is in black and WOR is in green. (d) Exponential distributions of pitch step sizes for adults (WR, black; WOR, green). (e) Age-dependent change in the exponential parameter λ of the WR (blue) and WOR (red) exponential pitch step size probability distributions for infants; (f) shows a similar plot for adults (WR, black; WOR, green). Note that only distributions that were determined to best fit to an exponential based on AIC criterion are represented in (a), (d), (e), and (f). (g) 90th percentiles of the infant pitch step size distributions plotted against infant age following adult response (WR, blue) and not (WOR, red); (h) shows a similar plot for adult pitch step sizes (WR, black; WOR, green). Medians and 90th percentile values were computed based on the raw data, prior to determining best fits using AIC.

For infants, as shown in Table S7 and Figure S7, we found a significant increase following adult response in the median pitch step size, suggesting that infants are more likely to take longer steps in the pitch dimension immediately after receiving adult responses. As infant age increased, median and 90th percentile pitch step sizes increased and λs of the exponential step size probability density fits decreased; these three findings suggest that as infants get older, they explore more broadly in the pitch dimension.

For adults, as shown in Table S7 and Figure S7, we found a significant decrease in 90th percentile value and a significant increase in λ parameter of the exponential fits following an infant response. Together, these results suggest that adults are more likely to take shorter steps following a response from an infant, indicating more focused exploration. We also found that median and 90th percentile values increased and λ decreased with infant age. These findings suggest that adults take longer steps in the pitch dimension as infant age increases, suggesting more adult pitch exploration and variation as the infant develops.

Finally, Figure S8 shows the median and 90th precentile values of pitch steps as a function of the number of vocalisation events by the vocaliser since a response was last received, for infant and adult vocalisations from the entire dataset (all recordings at all ages combined).

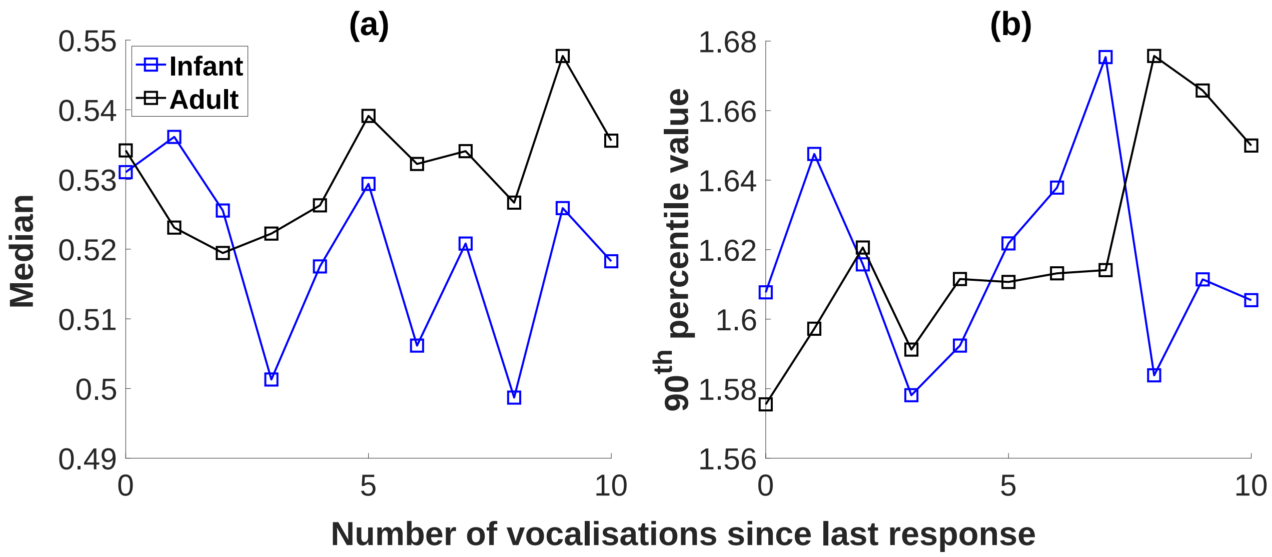

Figure S8: **Median and 90th percentile values for pitch steps as a function of number of steps since a response was last received.** (a) Median pitch step size for the entire dataset, as a function of number of vocalisations since a response was last received. The step from the vocalisation that received a response was assigned 0, the following step (assuming no response in the meantime) was assigned 1, and so on. This continued until the next response was received, at which point the count reset to 0. Infant data are shown in blue, and adult data are in black. (b) 90th percentile of pitch step sizes for the entire dataset, as a function of number of vocalisations since the last response was received (Infant, blue; adult, black). Note that plots were terminated after the 10th vocalisation event after the last response.

S3.3 Do step sizes in 2D acoustic space vary with response and infant age?

See main text for additional results and discussion.

For a demonstration of how lognormal and pareto distributions change as a function of their parameters, see `https://osf.io/2fuje/` (Wolfram Player may be used to view the demo). To see how the variation of the parameters of lognormal and pareto distributions in parameters regimes seen in our data per AIC best fits, see Fig. S9.

		Median	90th percentile	Lognormal parameter, μ	Lognormal parameter, σ
		**Infant vocalisations**			
**W/o sample size**	Response	-0.02 (p = 0.85)	-0.18 (p = 0.14)	-0.01 (p = 0.95)	**-0.40 (p < 0.001)**
	Infant age	0.08 (p = 0.16)	0.02 (p = 0.75)	0.05 (p = 0.40)	0.01 (p = 0.80)
		**Adult vocalisations**			
**W/o sample size**	Response	**0.24 (p = 0.01)**	0.02 (p = 0.83)	**0.26 (p = 0.003)**	**-0.60 (p < 0.001)**
	Infant age	**0.39 (p < 0.001)**	**0.30 (p < 0.001)**	**0.46 (p < 0.001)**	-0.15 (p = 0.01)

Table S8: **Steps in 2D acoustic space at the day-long recording level as a function of recent response and infant age: additional results of statistical analysis.** βs are shown with p-values in parentheses. Statistically significant results (at a significance level of 0.05) are in bold. Fixed effects are in rows and dependent variables are in columns. A separate linear mixed effects regression was run for each dependent variable. Infant ID was a random effect in all models. In the main text, sample size was included to control for possible co-variation between sample size and distribution fits, age, and response. Results when not controlling for sample size are shown here. The only difference in statistically significant relationships was observed for median infant step size increasing with infant age; this relationship only reached statistical significance when sample size was controlled. All values reported have been rounded to two decimal points wherever possible.

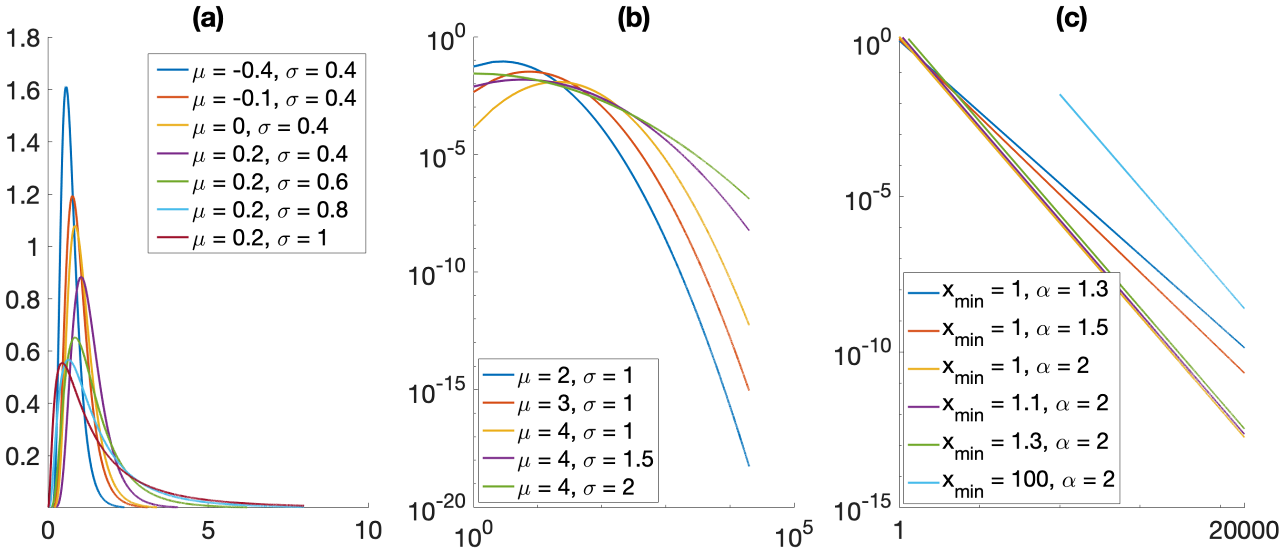

Figure S9: **Representative probability distributions to demonstrate how values affect fitted step size distribution shapes.** (a) has lognormal probability distributions for parameter ranges similar to those observed in AIC fits for infant and adult WR and WOR step size distributions in 2D acoustic space obtained from LENA and human-labelled data. As μ increases the peak widens while shifting to the right and the tail gets wider, making both intermediate steps and larger steps more likely. As σ increases, the peak widens while shifting to the left, and the tail widens, making shorter and larger steps more likely. (b) A similar plot for lognormal parameter ranges similar to those observed in AIC fits for WR and WOR temporal step size distributions (infants) obtained from LENA and human-labelled data. In contrast with plot (a), plot (b) uses log scales for both x and y axes, in order to better highlight the effects of differing parameter values in the ranges of interest. (c) A similar plot for pareto parameter ranges similar to those observed in AIC fits for adult WR and WOR temporal step size distributions obtained from LENA and human-labelled data. In addition, we add a reference curve at $x_{min} = 100$ to show the effect of increasing x_{min}. For the range of x_{min} values obtained from AIC best fits ($\sim$ 1-1.3) and the range of step sizes in time present in our data, the change in x_{min} has no appreciable effect on the pareto distribution. In contrast, however, as α increases, the distribution decays rapidly and the likelihood of larger step sizes decrease. For parameter ranges for infant and adult WR/WOR lognormal fits of step size distributions in 2D acoustic space from LENA-labelled data, see Fig. S10; for parameter ranges for infant and adult WR/WOR step size distributions in time from LENA-labelled data, see Fig. S12; for parameter values for human-labelled data and the corresponding LENA-labelled subset, see `https://osf.io/xptv4/` and `https://osf.io/56gx9/`. Note that the range of X-axis values used in (a), (b), and (c) correspond to the range of step size values for the data that went into the analyses reflected in Fig. S10 and S12. All probability distributions shown have been normalised such that the area under the curve from 0 to the maximum X-axis value shown, is 1.

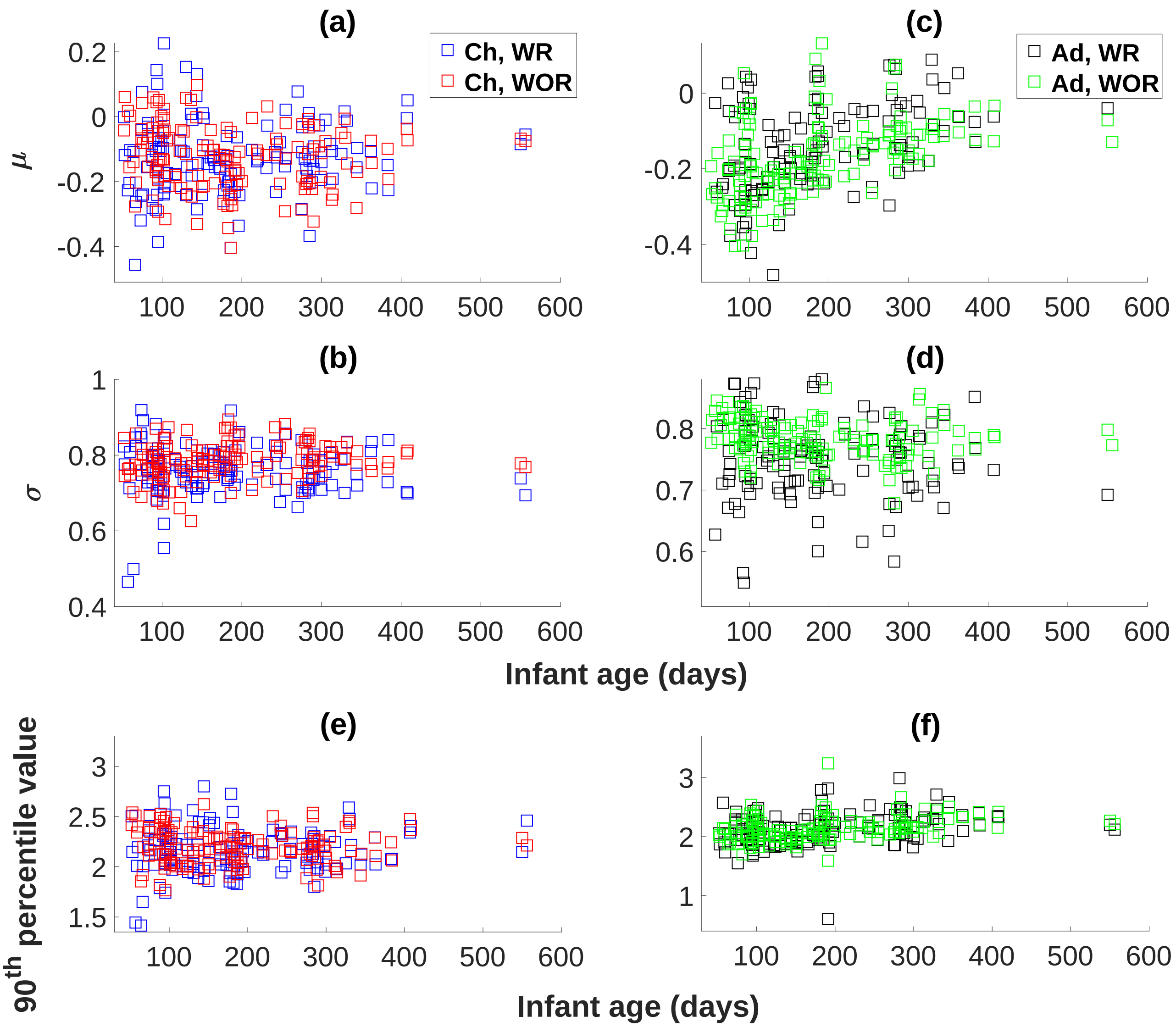

Figure S10: **Additional results of 2D acoustic space step size distribution analyses.** (a) Infant μ and (b) σ as a function of age and whether the child's first vocalisation received an adult response (WR, blue) or not (WOR, red). (c and d) show similar plots for adult (WR, black; WOR, green). Only distributions that were determined to best fit to lognormal based on AIC criterion are represented in (a-d). (e) 90th percentile values for step sizes in 2D acoustic space as a function of infant age for infants (WR, blue; WOR, red). (f) shows a similar plot for adults (WR, black; WOR, green). 90th percentile values were computed from the raw data, before finding the AIC best fit.

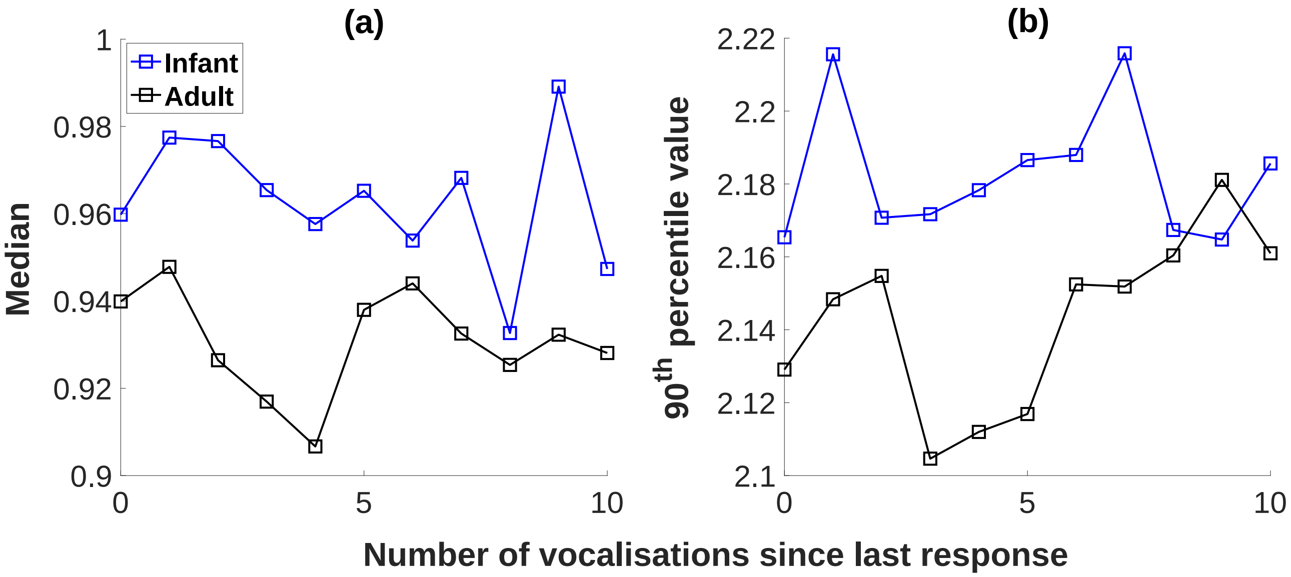

Figure S11: **Median and 90th percentile values for steps in 2D acoustic space as a function of number of events since last response.** (a) Median step size in 2D acoustic space for the entire dataset as a function of number of vocalisations since the last response was received. The step from the vocalisation that received a response was assigned 0, the following step (assuming no response in the meantime) was assigned 1, and so on. This continued until the next response was received, at which point the count reset to 0. The data for infants are in blue and data for adults are in black. (b) 90th percentile value of step sizes in 2D acoustic space for the entire dataset, as a function of number of vocalisations since the last response was received (infant, blue; adult, black). Note that plots were terminated after the 10th vocalisation event after the last response.

S3.4 Do inter-vocalisation intervals vary with response and infant age?

See main text for additional figures and results.

		Median	90th percentile	Lognormal parameter, μ	Lognormal parameter, σ
		**Infant vocalisations**			
**W/o sample size**	Response	**-0.25 (p = 0.02)**	-0.11 (p = 0.29)	-0.18 (p = 0.07)	**-0.42 (p < 0.001)**
	Infant age	-0.07 (p = 0.17)	**-0.13 (p = 0.02)**	**-0.15 (p = 0.004)**	**-0.17 (p = 0.001)**
		Median	90th percentile	Pareto parameter, x_{min}	Pareto parameter, α
		**Adult vocalisations**			
**W/o sample size**	Response	0.16 (p = 0.07)	-0.07 (p = 0.50)	**0.64 (p < 0.001)**	**0.33 (p < 0.001)**
	Infant age	**0.19 (p < 0.001)**	0.01 (p = 0.87)	-0.003 (p = 0.96)	**-0.21 (p < 0.001)**

Table S9: **Inter-vocalisation interval distributions at the day-long recording level as a function of recent response and infant age: results of statistical analysis.** βs are shown with p-values in parentheses. Statistically significant results (at a significance level of 0.05) are in bold. Fixed effects are in rows and dependent variables are in columns. A separate linear mixed effects regression was run for each dependent variable. Infant ID was a random effect in all models. In the main text, sample size was included to control for possible co-variation between sample size and distribution fits, age, and response. Here, results when not controlling for sample size are shown. When sample size was excluded, infant age showed a statistically significant negative relationship with the 90th percentile value and lognormal parameters σ and μ of infant inter-vocalisation interval distributions. Similarly, when sample size was included, the positive relationship between adult median inter-vocalisation interval and an infant response being recently received was only marginally significant. Finally, when sample size was included, we found a statistically significant negative effect in the μ parameter of infant inter-vocalisation step size distributions with respect to having recently received an adult response. All values reported have been rounded to two decimal points wherever possible.

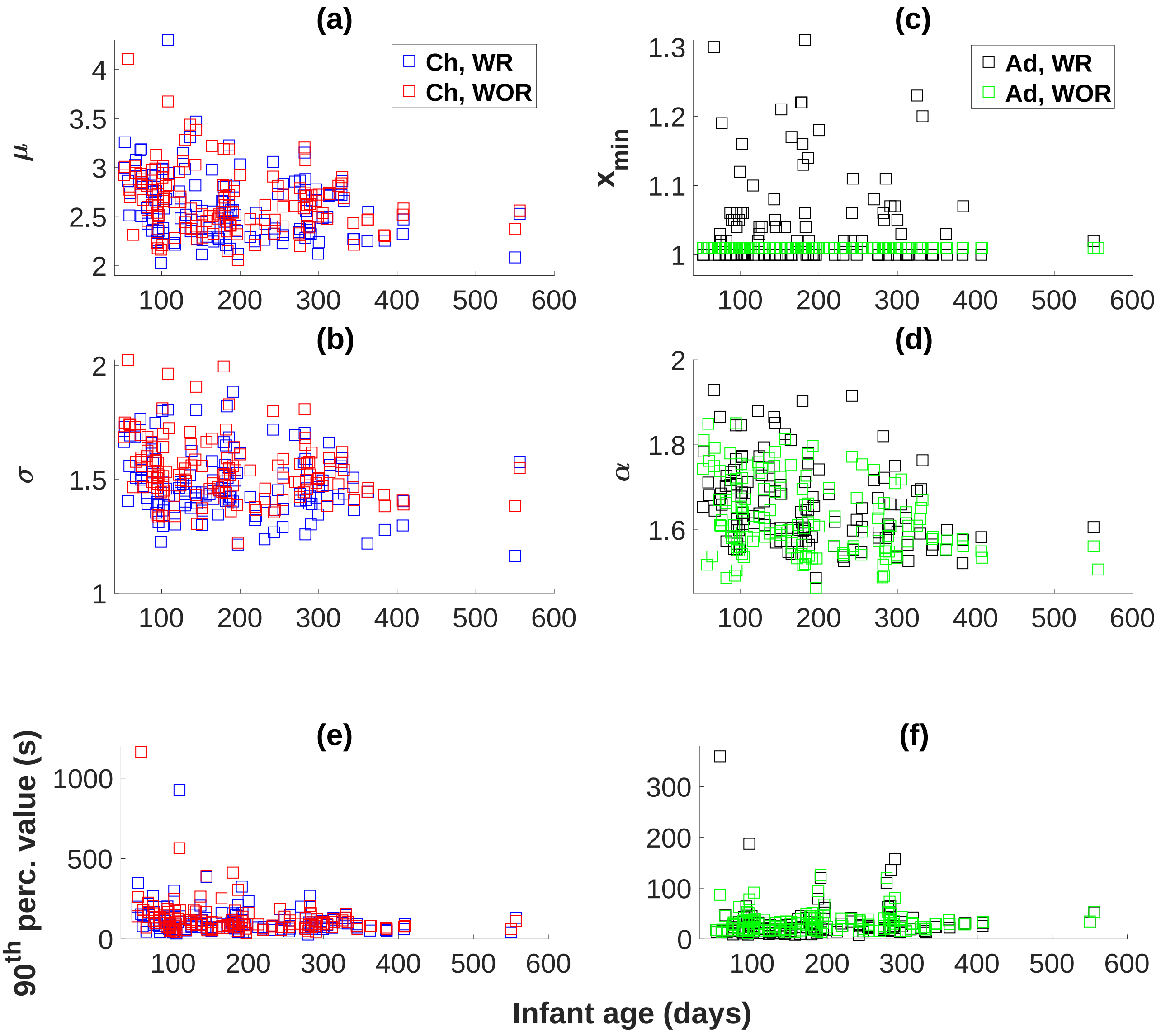

Figure S12: **Additional results of inter-vocalisation step size distribution analyses.** Infant μ (a) and σ (b) plotted against infant age following adult response (WR, blue) and not (WOR, red). Only distributions that were best fit to lognormal curves based on AIC are represented. Adult x_{min} (c) and α (d) plotted against infant age following infant response (WR, black) and not (WOR, green). Only distributions that were best fit to pareto curves per AIC are shown. (e) shows 90th percentile values for inter-vocalisation intervals plotted against infant age for infants (WR, blue; WOR, red). (f) shows a similar plot for adults (WR, black; WOR, green). 90th percentile values were computed from the raw data, before finding the AIC best fit.

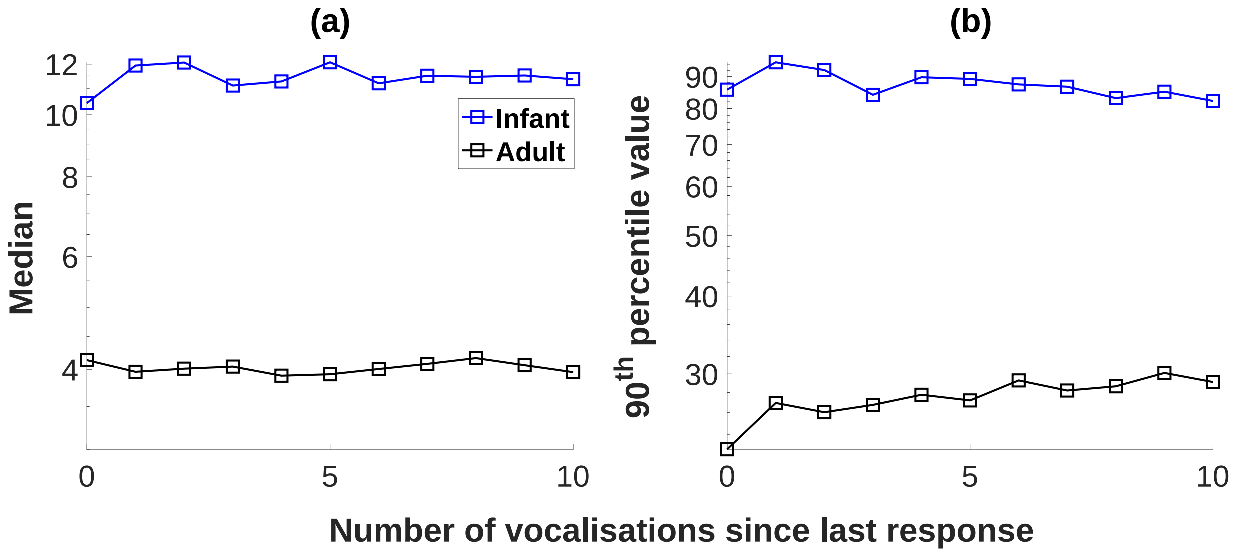

Figure S13: **Median and 90th percentile values for inter-vocalisation intervals as a function of number of events since a response was last received.** (a) Median inter-vocalisation interval for the entire dataset as a function of number of vocalisations since the last response was received. The step from the vocalisation that received a response to the next vocalisation was assigned 0, the following step (assuming no response in the meantime) was assigned 1, and so on. This continued until the next response was received at which point the count reset to 0. Data for infants are in blue and adult data are in black. (b) 90th percentile value of inter-vocalisation intervals for the entire dataset, as a function of number of vocalisations since the last response was received. Infant data is in blue and adult data is in black. Note that plots are terminated after the 10th vocalisation event after the last response.

Note that unlike other measures presented in this study (mean and standard deviation of pitch and standard deviation, median and 90th percentile values of step sizes in amplitude, pitch, and 2D acoustic space), infant inter-vocalisation intervals are longer than those of adults, based on median and 90th percentile values, i.e., infants vocalise more sparingly than adults. This observation is supported by Table S5. This disparity in median and 90th percentile values is in contrast to all other measures presented, which are comparable for both infants and adults.

S4. Using data re-labelled by human listeners to check the validity of automatically labelled data

S4.1 Inter-rater reliability measures

	Ch only (H)	Ad only (H)	Ch+Olp; no Ad (H)	Ad+Olp; no Ch (H)	Ch/Ad (H)	No Ch/Ad (H)
**L1, 274**						
Ch (L)	0.49	0.05	0.61	0.10	0.83	0.17
Ad (L)	0.08	0.32	0.11	0.45	0.87	0.13
**L1, 340**						
Ch (L)	0.67	0.04	0.72	0.08	0.86	0.14
Ad (L)	0.02	0.74	0.02	0.89	0.96	0.04
**L3, 340**						
Ch (L)	0.72	0.04	0.78	0.06	0.90	0.09
Ad (L)	0.02	0.75	0.02	0.87	0.96	0.04
**L2, 530**						
Ch (L)	0.51	0.11	0.57	0.15	0.83	0.17
Ad (L)	0.14	0.37	0.16	0.43	0.74	0.26

Table S10: **Fraction of LENA speaker labels as identified by human listeners.** The fraction of infant speech-related (CHNSP) labels and adult (MAN or FAN) labels as labelled by LENA that fall into various label categories as determined by human listeners is reported, for each human-labelled dataset. The human-labelled categories include infant speaker label (CHN) only (Ch only, column 2); adult (MAN or FAN or both) speaker only (Ad only, column 3); infant speaker with possible other child overlap but no adult overlap (column 4); adult speaker with possible other child (not the infant wearing the recorder) overlap (column 5); infant (CHN) and/or adult (MAN or FAN) speakers with possible overlap of other child voice (column 6); and either no voices identified or other child identified, but no target infant (CHN) or adult (MAN or FAN). For a description of human-listener vs. LENA labels, refer to the Methods section of the main text. All values reported have been rounded to two decimal points wherever possible. We see fairly good agreement ($\sim 70\%$ or greater) between human listeners and LENA for the infant 340 data labelled by listeners 1 and 3 both when overlaps are allowed (Adult (L) vs. column 5, and Ch (L) vs. column 4) and not allowed (Adult (L) vs. column 3, and Ch (L) vs. column 2). Note that these are also the human-labelled datasets with the highest percent agreement and Cohen's Kappa values (Table 3, main text). These values are lower for data from infant 274 labelled by listener 1, and data from infant 530 labelled by listener 2, both of which have lower inter-rater reliability scores. In addition, the data loss as a consequence of more restrictions on determining what is an infant vocalisation (CHN only vs. CHN+Olp; no Ad vs. Ch/Ad) or an adult vocalisation (Ad only vs. Ad+Olp; no Ch vs. Ch/Ad) for human-labelled data can be seen clearly in the table.

	CHN (H)	FAN (H)	MAN (H)		CHN (H)	FAN (H)	MAN (H)
**L1, 274**				**L1, 340**			
CHNSP (L)	320	32	0	CHNSP (L)	462	12	17
FAN (L)	99	295	12	FAN (L)	23	269	137
MAN (L)	22	26	83	MAN (L)	3	17	512
**L2, 530**				**L3, 340**			
CHNSP (L)	954	195	12	CHNSP (L)	502	12	16
FAN (L)	278	445	113	FAN (L)	24	258	147
MAN (L)	66	12	287	MAN (L)	3	15	541

Table S11: **Confusion matrices for LENA vs. human-listener labelled data.** Confusion matrices for each set of human-labelled data are shown, with LENA labels as the known class (row indices) and human-listener labels as the predicted class (column indices). For a description of human-listener vs. LENA labels, refer to the Methods section of the main text.

**Infant 340**	CHN (L3)	FAN (L3)	MAN (L3)
CHN (L1)	478	0	0
FAN (L1)	0	266	10
MAN (L1)	1	0	631

Table S12: **Confusion matrix for human listeners 1 vs. 3 for data from infant 340.** Confusion matrix for data from infant 340 labelled by listeners 1 and 3 is shown, with L1 labels as the known class (row indices) and L3 labels as the predicted class (column indices). For a description of human-listener labels, refer to the Methods section of the main text. Note that we see high agreement between L1 and L3 labels, which is in agreement with the high inter-rater reliability scores for listeners 1 and 3 for data from infant 340 (see Table 3, main text).

S4.2 Acoustic space trajectories and step size distributions of infants and adults

In Figures S14, S15, S16, and S17, we present data from three infants at different ages re-labelled by human listeners. For comparison, we also present the corresponding data (i.e. from the same day-long recording) as labelled by the LENA software. In addition to comparing how data labelled by the LENA software compares to the same data labelled by human listeners, we have one recording (participant 340, age 183 days) labelled by two different human listeners to compare how differences in labelling by different listeners affect the data.

In each of the four figures, panel (a) shows infant vocalisations' locations in 2-D acoustic space and panel (b) shows adult vocalisations' locations in the same space. The data are depicted as a series of directed vectors from vocalisation i at a location in the acoustic space given by the ordered pair (f_i, d_i) to vocalisation $i+1$ at (f_{i+1}, d_{i+1}), starting from the first available vocalisation based on the data. Here, f is the z-scored log pitch, and d is the z-scored amplitude. Pink vectors start at infant vocalisations that have received adult responses (corresponding to WR steps) and grey vectors start at infant vocalisations that have not received responses (corresponding to WOR steps), as labelled by the human listener. These plots are on the left side of the (a) panels. On the left side of panel (b), pink vectors start at adult vocalisations that have received infant responses (WR steps) and grey vectors start at adult vocalisations that have not received infant responses (WOR steps), as labelled by the human listener. On the right side of panel (a) are infant step data based on LENA labels, with blue vectors starting at infant vocalisations that have received adult responses (WR steps) and red vectors starting at infant vocalisations that have not received responses (corresponding to WOR steps). Finally, on the right side of panel (b) are the adult step data based on LENA labels, with black vectors starting at adult vocalisations that have received infant responses (WR steps) and green vectors starting at adult vocalisations that have not received responses (WOR steps). Note that vocalisations that were marked as 'not applicable' for whether a response was received or not were excluded from these plots.

Finally, we present raw and fitted probability distributions of steps in pitch, amplitude, 2D acoustic space, and time. Each plot shows a set of human-labelled data together with the same recording's data as labelled by the LENA software. Plots c1–c4 and d1–d4 show raw probability distributions of step sizes following response (WR) based on human labelled utterances (pink) and the corresponding data as labelled by LENA (infant: blue; adult: black). Plots c5–c8 and d5–d8 show raw probability distributions of step sizes following no response (WOR) for human labelled utterances (grey) and the corresponding data labelled by LENA (infant: red; adult: green). Plots e1–f8 show best fit curves for step size probability distributions from human labelled data and the corresponding data labelled by LENA. Dashed pink lines are for human-labelled data WR (where the first vocalisation received a response), dashed grey are for human-labelled data WOR (where the first vocalisation did not receive a response), dashed blue lines are for the corresponding LENA-labelled WR data for infants, and dashed black lines are for the corresponding LENA-labelled adult WR data. Similarly, dashed red lines are LENA-labelled infant WOR data and dashed green lines are LENA-labelled adult WOR data. More details about the fits and fit parameters available here: `https://osf.io/8ern6/`, in folder Reported_and_auxiliary_results.

We see that the without response (WOR) steps' distributions are extremely similar when LENA's labels vs. human re-labelling are used. On the other hand, for with-response steps, we see larger differences between the distribution fits (shown in dashed lines in plots e1–e4 and f1–f4) for human-labelled vs. LENA-labelled data. We also see that the raw distributions are much less smooth for human-labelled with-response data. A likely reason for these discrepancies is the paucity of with-response data in the human-labelled dataset (see Table S13). This in turn may perhaps be due to human listeners' greater sensitivity to voices, so that segments labelled by humans may have been more likely to be identified as containing multiple human voices and therefore excluded from analysis. Additionally, exclusion of many of segments of the audio (based on LENA's automatic segmentation and classification) from the human labelling task made it unlikely that relevant voices that were missed by the LENA system would have been included in the human analysis. For all these reasons, responses were less likely to be identified within the human-labelled datasets. For quantitative results on how step size distributions based on human-labelled data compare to those based in corresponing LENA-labelled data, see Table S14.

Child ID and age	LENA Ad		LENA Ch		Listener ID	HUM Ad		HUM Ch	
	WR	WOR	WR	WOR		WR	WOR	WR	WOR
274, 82 days	100	941	124	383	L1	7	359	10	338
340, 183 days	76	1001	86	438	L1	7	771	10	397
340, 183 days	76	1001	86	438	L3	15	781	13	425
530, 95 days	295	1769	340	1289	L2	7	492	17	1060

Table S13: **Number of data points in WR and WOR step sizes for human re-labelled data and corresponding LENA data.** The number of data points for both WR and WOR step sizes for all three datasets that were re-labelled by human listeners are shown. These numbers are reported for adult vocalisations (Ad) and infant vocalisations (Ch), for both human re-labelled data (HUM) and the corresponding LENA data (LENA). The LENA-labelled data include many more steps for all types except child steps in which the first vocalisation was not followed by an adult response – we find that the number of steps for this category is comparable for both human-labelled data and corresponding LENA-labelled data.

Infant ID, age, and listener ID	f_{rej}						
Infant 274, 82 days, listener L1	0.83	Infant (unsplit)	0.37 (0.35)	Infant (WR)	0.33 (0.32)	Infant (WOR)	0.46 (0.29)
		Adult (unsplit)	0.18 (0.13)	Adult (WR)	0.51 (0.39)	Adult (WOR)	0.28 (0.19)
Infant 340, 183 days, listener L1	0.92	Infant (unsplit)	0.35 (0.35)	Infant (WR)	0.79 (0.13)	Infant (WOR)	0.39 (0.39)
		Adult (unsplit)	0.74 (0.49)	Adult (WR)	0.76 (0.36)	Adult (WOR)	0.73 (0.49)
Infant 340, 183 days, listener L3	0.92	Infant (unsplit)	0.69 (0.22)	Infant (WR)	0.65 (0.12)	Infant (WOR)	0.70 (0.31)
		Adult (unsplit)	0.67 (0.45)	Adult (WR)	0.63 (0.33)	Adult (WOR)	0.69 (0.46)
Infant 530, 95 days, listener L2	0.46	Infant (unsplit)	< 0.001 (< 0.001)	Infant (WR)	0.51 (0.36)	Infant (WOR)	0.02 (0.02)
		Adult (unsplit)	0.05 (0.05)	Adult (WR)	0.65 (0.35)	Adult (WOR)	0.25 (0.42)

Table S14: **Two-sample Kolmogorov-Smirnov (KS2) test results, comparing step size distributions from human-labelled data and corresponding LENA labelled data.** f_{rej} represents the fraction of tests which failed to reject the null hypothesis (that data from the two samples – step size distriution from data labelled by LENA and human listeners – are drawn from the same distribution) at the 0.05 significance level, for individual datasets (column 1). For each category specified in columns 3, 5, and 7, we present the mean p-value from the KS2 test in columns 4, 6, and 8, respectively. The standard deviation of the p-value in parentheses. For example, for data from infant 274 at 82 days labelled by listener 1, 83 percent of KS2 tests (performed on unsplit, WR, and WOR step size distributions in pitch, amplitude, 2D acoustic space, and time, for infants and adults) failed to reject the null hypothesis. Further, for all unsplit step size distributions (pitch, amplitude, 2D acoustic space, and time) where the infant was the vocaliser, the mean p-value associated with the KS2 tests performed was 0.37, with a standard deviation of 0.35. By and large, we see that f_{rej} is high for all datasets except infant 530 at 95 days labelled by listener 2. Note that f_{rej} is lowest for data from infant 530 at 95 days labelled by listener 2, followed by data from infant 274 at 82 days labelled by listener 1, both of which have the lowest reliability scores (Table 3, main text; subsection S4.1). Similarly, we also see the lowest mean p-values for these datasets. All values reported have been rounded to two decimal points wherever possible. For more detailed results, see `https://osf.io/8ern6/` .

Labelled by	Vocalisation	Step type	Mean R^2	Std. dev	Mean observations per distribution	Number of distributions
HUM	Infant	Unsplit	0.90	0.06	684	16
LENA (subset)	Infant	Unsplit	0.94	0.03	1188.67	12
HUM	Infant	WOR	0.90	0.09	555	16
LENA (subset)	Infant	WOR	0.94	0.04	703.33	12
HUM	Infant	WR	0.22	0.24	12.5	16
LENA (subset)	Infant	WR	0.69	0.21	183.33	12
HUM	Adult	Unsplit	0.90	0.06	861.5	16
LENA (subset)	Adult	Unsplit	0.93	0.03	1930.33	12
HUM	Adult	WOR	0.90	0.09	600.75	16
LENA (subset)	Adult	WOR	0.94	0.06	1237	12
HUM	Adult	WR	0.18	0.24	9	16
LENA (subset)	Adult	WR	0.69	0.22	157	12

Table S15: **Goodness of AIC fits (human-labelled data and corresponding LENA-labelled subset).** The means and standard deviations of the R^2 value of the AIC best fit for different step size distribution types are shown. All results are from data labelled by human listeners and the corresponding LENA-labelled subset. The step size distributions are organised by whether they were computed from data where the vocaliser was an infant or adult (column 1), and whether they are WOR, WR, or unsplit distributions (column 2). For mean and standard deviation for R^2 values for each step size distribution type for a category (eg. WR pitch step size distributions of adult vocalisations), see `https://osf.io/53amv/`. For a breakdown of the majority best fit for each distribution type, see Fig. S4. The sixth column has the mean number of observations per distribution for that category while the seventh column has the total number of distributions that went into calculating the mean and standard deviation of R^2 values for that category. For example, the first row of the table gives the mean and standard deviation of all unsplit step size distributions (pitch, amplitude, 2d acoustic space, and time) where the vocaliser was an infant as labelled by human listeners, regardless of best fit type. For this category, each distribution on average had 684 observations, and 16 distributions were used to calculate the mean and standard deviation R^2 values. All values reported have been rounded to two decimal points wherever possible. R^2 values typically fall between 0 to 1, with values closer to 1 indicating better fits. Note that one possible reason for lower R^2 values could be that some step types were less prevalent and therefore had fewer steps on which to fit the distribution (see the sixth column of the table).

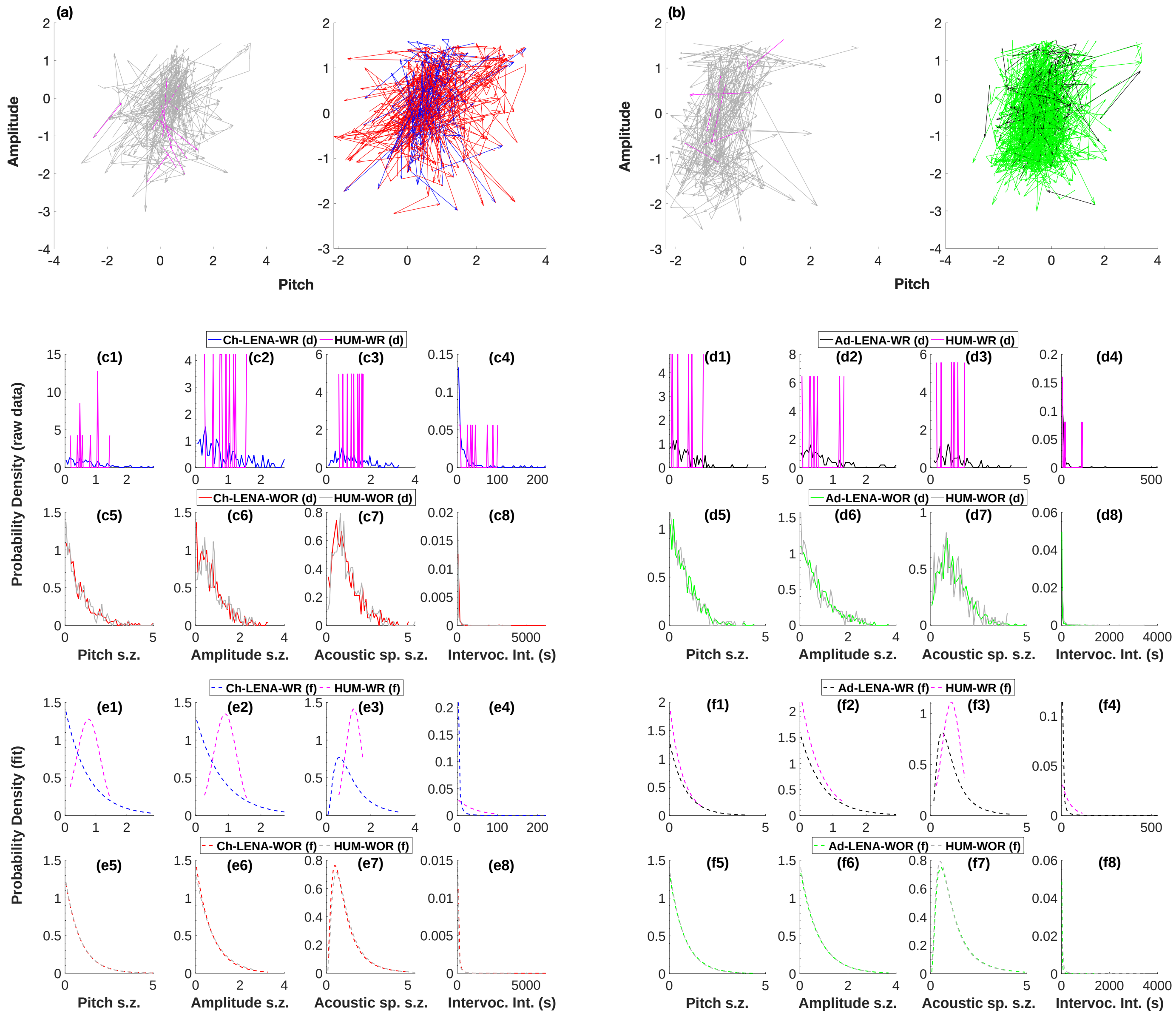

Figure S14: **Human labelled data and corresponding LENA labelled data - Participant 274 at 82 days old; Listener 1.** (a) Acoustic space traversed by the infant as labelled by human listener L1 (WR in pink and WOR in grey; left), and as labelled by the LENA software (WR in blue and WOR in red; right). (b) Acoustic space traversed by the adult as labelled by human listener L1 (WR in pink and WOR in grey; left), and as labelled by the LENA software (WR in black and WOR in green; right). Raw and fitted probability distributions of step sizes from human labelled data and corresponding LENA labelled data are shown in panels (c1) through (f8). Infant data for step size distributions following a response (WR) are in panels c1–c4 and infant data for WOR are in panels c5–c8. Adult WR data are in panels d1–d4, and adult WOR data are in panels d5–d8. Fits for Infant WR data are in panels e1–e4 and infant WOR fits are in panels e5–e8. Adult WR fits are in panels f1–f4, and adult WOR fits are in panels f5–f8. The (f) in the legend indicates fitted as opposed to raw data (indicated by (d) in the legend). Note that both human-labelled infant and adult WR and WOR data/fits are given by pink and grey solid/dashed lines and are presented in the same subplots as their corresponding LENA-labelled data/fits, to allow for visual comparison of how well the curves do, or in a few cases do not, overlap.

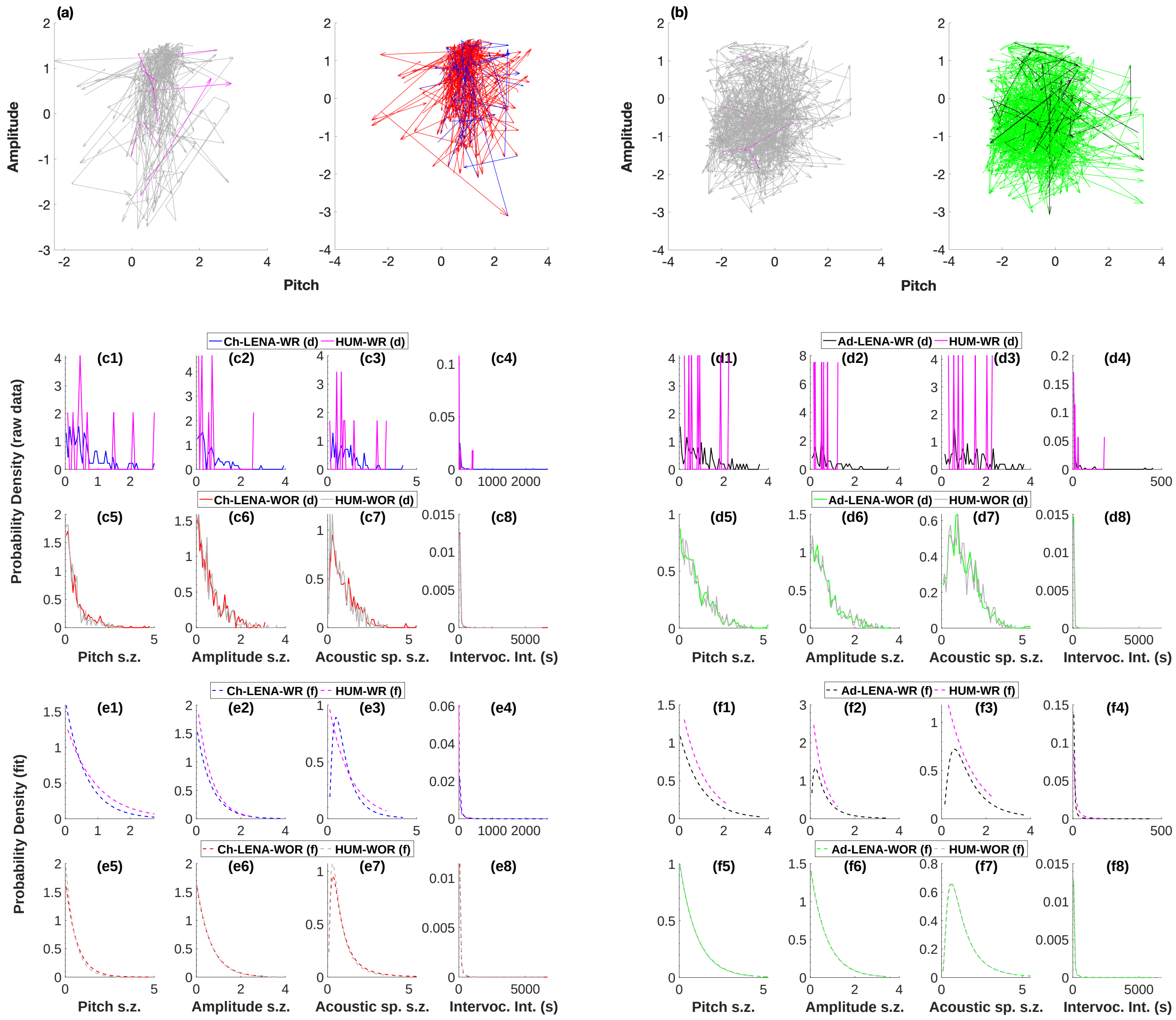

Figure S15: **Human labelled data and corresponding LENA labelled data - Participant 340 at 183 days old; Listener 1.** (a) Acoustic space traversed by the infant as labelled by human listener L1 (WR in pink and WOR in grey; left), and as labelled by the LENA software (WR in blue and WOR in red; right). (b) Acoustic space traversed by the adult as labelled by human listener L1 (WR in pink and WOR in grey; left), and as labelled by the LENA software (WR in black and WOR in green; right). Raw and fitted probability distributions of step sizes from human labelled data and corresponding LENA labelled data are shown in panels (c1) through (f8). Infant data for step size distributions following a response (WR) are in panels c1–c4 and infant data for WOR are in panels c5–c8. Adult WR data are in panels d1–d4, and adult WOR data are in panels d5–d8. Fits for Infant WR data are in panels e1–e4 and infant WOR fits are in panels e5–e8. Adult WR fits are in panels f1–f4, and adult WOR fits are in panels f5–f8. The (f) in the legend indicates fitted as opposed to raw data (indicated by (d) in the legend). Note that both human-labelled infant and adult WR and WOR data/fits are given by pink and grey solid/dashed lines and are presented in the same subplots as their corresponding LENA-labelled data/fits, to allow for visual comparison of how well the curves do, or in a few cases do not, overlap.

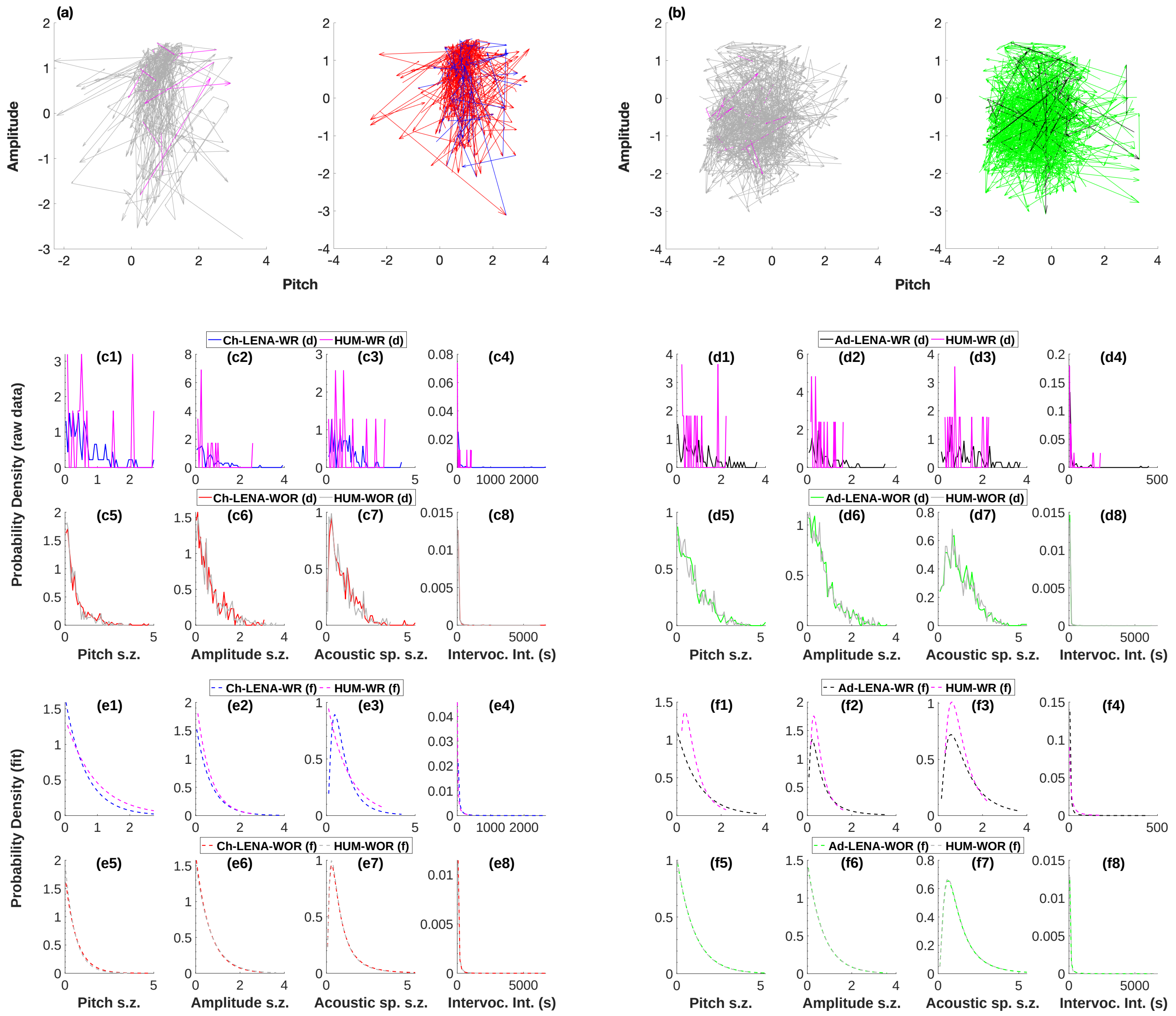

Figure S16: **Human labelled data and corresponding LENA labelled data - Participant 340 at 183 days old; Listener 3** (a) Acoustic space traversed by the infant as labelled by human listener L3 (WR in pink and WOR in grey; left), and as labelled by the LENA software (WR in blue and WOR in red; right). (b) Acoustic space traversed by the adult as labelled by human listener L3 (WR in pink and WOR in grey; left), and as labelled by the LENA software (WR in black and WOR in green; right). Raw and fitted probability distributions of step sizes from human labelled data and corresponding LENA labelled data are shown in panels (c1) through (f8). Infant data for step size distributions following a response (WR) are in panels c1–c4 and infant data for WOR are in panels c5–c8. Adult WR data are in panels d1–d4, and adult WOR data are in panels d5–d8. Fits for Infant WR data are in panels e1–e4 and infant WOR fits are in panels e5–e8. Adult WR fits are in panels f1–f4, and adult WOR fits are in panels f5–f8. The (f) in the legend indicates fitted as opposed to raw data (indicated by (d) in the legend). Note that both human-labelled infant and adult WR and WOR data/fits are given by pink and grey solid/dashed lines and are presented in the same subplots as their corresponding LENA-labelled data/fits, to allow for visual comparison of how well the curves do, or in a few cases do not, overlap.

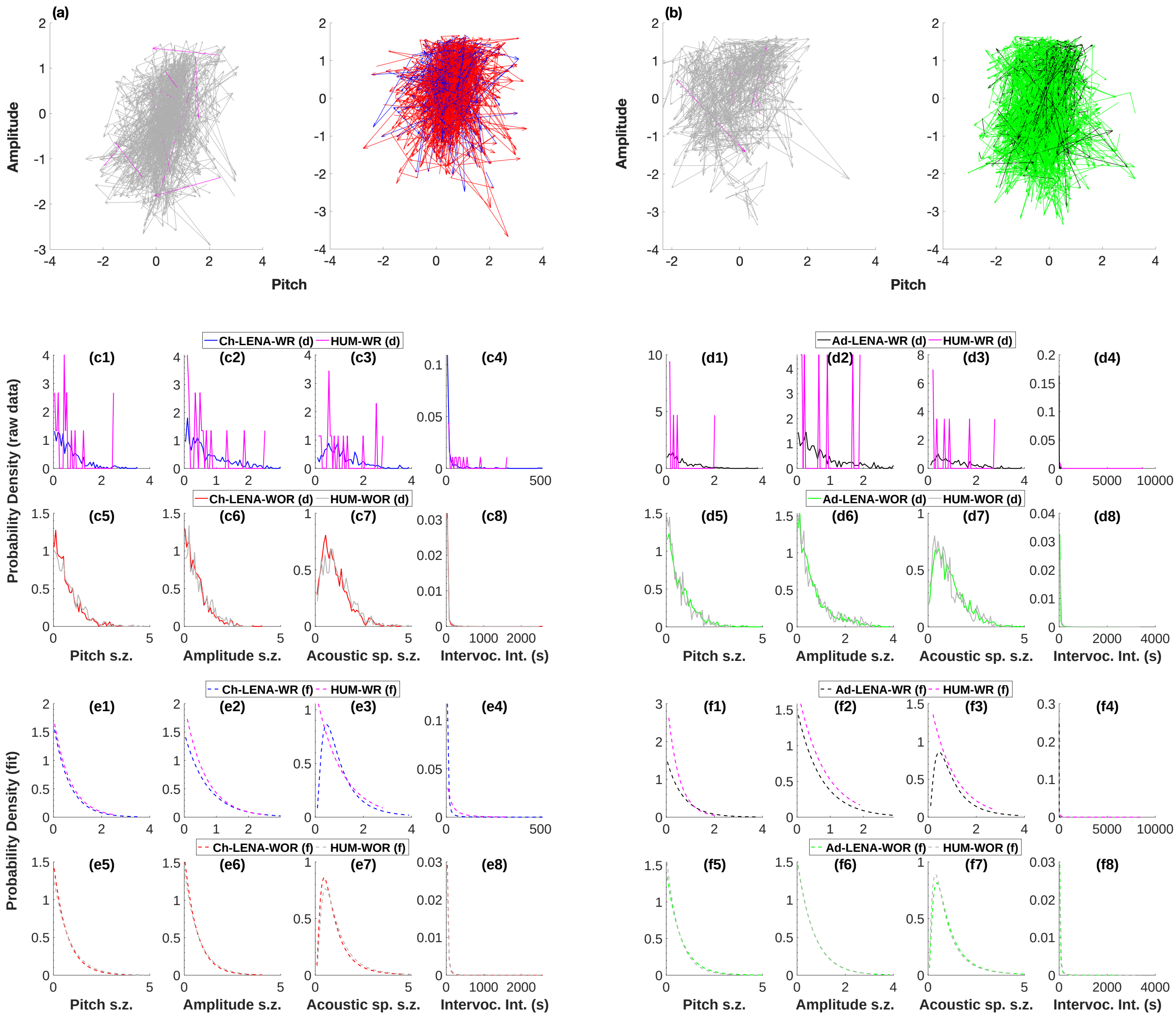

Figure S17: **Human labelled data and corresponding LENA labelled data - Participant 530 at 95 days old; Listener 2** (a) Acoustic space traversed by the infant as labelled by human listener L2 (WR in pink and WOR in grey; left), and as labelled by the LENA software (WR in blue and WOR in red; right). (b) Acoustic space traversed by the adult as labelled by human listener L2 (WR in pink and WOR in grey; left), and as labelled by the LENA software (WR in black and WOR in green; right). Raw and fitted probability distributions of step sizes from human labelled data and corresponding LENA labelled data are shown in panels (c1) through (f8). Infant data for step size distributions following a response (WR) are in panels c1–c4 and infant data for WOR are in panels c5–c8. Adult WR data are in panels d1–d4, and adult WOR data are in panels d5–d8. Fits for Infant WR data are in panels e1–e4 and infant WOR fits are in panels e5–e8. Adult WR fits are in panels f1–f4, and adult WOR fits are in panels f5–f8. The (f) in the legend indicates fitted as opposed to raw data (indicated by (d) in the legend). Note that both human-labelled infant and adult WR and WOR data/fits are given by pink and grey solid/dashed lines and are presented in the same subplots as their corresponding LENA-labelled data/fits, to allow for visual comparison of how well the curves do, or in a few cases do not, overlap.

S5. What vocalisation acoustics and changes in vocalisation acoustics predict responses?

	**Probability of infant receiving adult response**	**Probability of adult receiving infant response**
Infant age	**-0.16 ($p < 0.001$)**	-0.01 ($p = 0.08$)
Pitch	**-0.32 ($p < 0.001$)**	**0.24 ($p < 0.001$)**
Amplitude	**0.18 ($p < 0.001$)**	**0.44 ($p < 0.001$)**
Pitch step	**-0.02 ($p = 0.002$)**	**-0.07 ($p < 0.001$)**
Amplitude step	**0.03 ($p < 0.001$)**	**0.09 ($p < 0.001$)**
Time step	**-0.07 ($p < 0.001$)**	**-0.03 ($p = 0.02$)**

Table S16: **Which vocalisation patterns received responses: results of statistical analysis with patterns of change in acoustics included.** βs are given with p-values in brackets. Significant results (at a significance level of 0.05) are in bold. The 'step' variables are the step sizes from the preceding vocalisation to the vocalisation in question. Note that acoustic step sizes are non-directional and may represent either increasing or decreasing amplitude or pitch. Infant ID was a random effect in all models. All values reported have been rounded to two decimal points wherever possible.
